# Notch Signalling Synchronizes the Zebrafish Segmentation Clock but Is Not Needed To Create Somite Boundaries

**DOI:** 10.1371/journal.pgen.0040015

**Published:** 2008-02-01

**Authors:** Ertuğrul M Özbudak, Julian Lewis

**Affiliations:** Vertebrate Development Laboratory, Cancer Research UK London Research Institute, London, United Kingdom; University of Pennsylvania School of Medicine, United States of America

## Abstract

Somite segmentation depends on a gene expression oscillator or clock in the posterior presomitic mesoderm (PSM) and on read-out machinery in the anterior PSM to convert the pattern of clock phases into a somite pattern. Notch pathway mutations disrupt somitogenesis, and previous studies have suggested that Notch signalling is required both for the oscillations and for the read-out mechanism. By blocking or overactivating the Notch pathway abruptly at different times, we show that Notch signalling has no essential function in the anterior PSM and is required only in the posterior PSM, where it keeps the oscillations of neighbouring cells synchronized. Using a GFP reporter for the oscillator gene *her1*, we measure the influence of Notch signalling on *her1* expression and show by mathematical modelling that this is sufficient for synchronization. Our model, in which intracellular oscillations are generated by delayed autoinhibition of *her1* and *her7* and synchronized by Notch signalling, explains the observations fully, showing that there are no grounds to invoke any additional role for the Notch pathway in the patterning of somite boundaries in zebrafish.

## Author Summary

The somites—the embryonic segments of the vertebrate body—form one after another from tissue at the tail end of the embryo. A gene expression oscillator, the somite segmentation clock, operating in this tail region, marks out a periodic spatial pattern and so controls the segmentation process. Evidence from mutants shows that the Notch cell-cell signalling pathway has a critical role in the clock mechanism. However, when we switch on a blockade of Notch signalling, by immersing zebrafish embryos in the chemical inhibitor DAPT, the next ∼12 somites form normally, and only after that do disrupted somites appear. We show that this is because Notch signalling is needed only to maintain synchrony between the clocks of individual cells. The cells take about seven cycles to drift out of synchrony when Notch-mediated communication is blocked, and then a further five cycles to pass from the site where the tissue receives its “time-stamp” to the site where overt segmentation begins. By mathematical modelling, backed up with measurements on transgenic embryos, we show how Notch signalling may act at a molecular level to synchronise the intracellular oscillators of adjacent individual cells.

## Introduction

The segments of the vertebrate trunk and tail originate from a series of blocks of tissue, the somites, that are formed on each side of the body axis during early development. Mutations in the Notch cell-cell signalling pathway disrupt somite formation and lead to an irregularly segmented body axis [1–14]. But how do they cause this effect? What specific part does Notch signalling play in creating the regular somite pattern?

At least three different answers have been suggested. All of them start from the observation that the spatially periodic pattern of somites and intersomitic clefts is laid down sequentially through a temporally periodic clock-like process operating at the tail end of the embryo, in the presomitic mesoderm (PSM). Cells in the PSM show oscillating expression of several genes, with neighbouring cells oscillating in synchrony. The oscillating genes include *Hes1*, *Hes7*, *Lfng*, *Snail1,* and *Axin2* in the mouse, *Hes1*, *c-hairy2*, *Lfng*, and *Snail2* in the chick, and *her1*, *her7*, and *deltaC* in the zebrafish [1,15–25]. An FGF signal originating in the tail bud is thought to define the extent of the PSM [26], keeping cells that are within range of this signal in an active, plastic state in which they not only show oscillating gene expression but also continue to proliferate [27]. As a result of the proliferation, there is a continual overflow of cells from the anterior end of the PSM; here, the emerging cells cease oscillating and rearrange their contacts to form visible somites. The segmentation clock runs at its full speed in the cells in the posterior part of the PSM, and this dictates the periodicity of the whole process of somite formation: the set of cells emerging from the PSM in the course of one such clock cycle constitute precisely one somite.

As successive groups of PSM cells pass from the posterior into the anterior part of the PSM, their oscillations slow down in preparation for exit from the PSM [25,28,29]; at the same time, the cells switch on expression of additional genes which, in a manner not yet fully understood, create the final segmentation pattern that becomes manifest as the cells at last emerge from the PSM and halt their oscillations (reviewed in [14,26,30]). These additional genes operating in the anterior part of the PSM in effect read the phase of oscillation of each cohort of cells as they enter the anterior PSM and stamp the successive cohorts with different final characters according to their clock phase at the time of entry into that region [28]. The somite segmentation pattern created in this way represents a spatial trace of the oscillations of the segmentation clock.

Mutations in the Notch pathway disrupt the organized temporal pattern of oscillations in the posterior PSM [1,2,4,15–17,23,24,31–33]. One proposal, therefore, is that Notch signalling is required to generate the gene expression oscillations in each cell in the PSM [2,16,34–36]. We shall call this the *oscillation-generator hypothesis*.

Notch pathway mutations also disrupt the pattern of expression of the read-out genes in the anterior PSM. Hence, another suggestion is that Notch signalling has an additional role at a later stage, while cells are in the anterior part of the PSM, where it could be required to create the sharp boundaries of gene expression that demarcate each new somite [37–43], just as it is required in the insect wing disk to create the sharp boundary between dorsal and ventral compartments [44]. We shall call this the *boundary-formation hypothesis*.

Lastly, Notch pathway mutations lead to a loss of coordination between neighbouring cells in the PSM, so that the tissue appears as a pepper-and-salt mixture of cells expressing the oscillator genes at different levels. This has led to a third hypothesis: that cell–cell communication via the Notch pathway is required to keep the oscillations of neighbouring cells synchronized [15,27]. We shall call this the *synchronization hypothesis*.

These three hypotheses are not mutually exclusive, and in principle they could all be true. Indeed, persuasive evidence has been adduced in favour of each of them, although this evidence comes from observations in different vertebrate species in the different cases. In the chick, blocking Notch signalling with the γ-secretase inhibitor DAPT, for example, leads to a failure of oscillation of the clock gene *Lfng*, or at least a drastic reduction of its amplitude [34], favouring the oscillation-generator hypothesis. In the mouse, sharp boundaries in the level of activation of Notch foreshadow the formation of intersomitic clefts, favouring the boundary-formation hypothesis [41–43]. And in zebrafish, the pepper-and-salt pattern of expression of the clock gene *deltaC* in Notch pathway mutants supports the synchronization hypothesis [15]. This last hypothesis also fits the curious observation that mutations in Notch pathway components, while disrupting the segmentation of posterior somites, generally allow the first four to eight somites to develop normally [13,15]: if PSM cells in a mutant all start their oscillations in synchrony when the PSM is first established during gastrulation, but then take four to eight oscillator cycles to drift out of synchrony, such a result is expected [15]. Recent papers on zebrafish somitogenesis have added to the evidence for the synchronization hypothesis. Thus Horikawa et al. [27] have used genetic mosaics to show that the oscillations of neighbouring cells in the PSM are coupled via the Notch pathway, and have also demonstrated that blocking Notch signalling with the inhibitor DAPT leads to loss of synchrony. Giudicelli et al. [28] have tested the predictions of a mathematical model of the proposed synchronization mechanism [29] and have shown that the measured delays in the pathway are consistent with the model. Mara et al. [45], on the basis of a complex set of experiments with mutants and inhibitors, have argued that signalling via the Notch ligand DeltaC serves to synchronize oscillations in the PSM. Most recently, Riedel-Kruse et al. [46] (in a study published after the present paper was submitted) have studied the effects of inhibiting Notch signalling to varying degrees, starting before the onset of somite segmentation, and have presented a mathematical model illustrating how the resulting variation in the level of onset of somite defects can be explained on the basis of the synchronization hypothesis. These recent papers, however, while supporting the synchronization hypothesis, still do not put forward arguments to exclude the oscillation-generator hypothesis or the boundary-formation hypothesis.

Are all three ideas then correct and true of somite development for vertebrates in general? In this paper, we argue that the answer is no: in the zebrafish, at least, Notch signalling is required for one and only one function in somite segmentation—to keep the oscillations of neighbouring PSM cells synchronized. A simple mathematical model of the segmentation clock mechanism explains how Notch signalling can exert its synchronizing influence. Using a reporter transgene, we measure the influence of Notch signalling on the mean level of clock gene expression; the effect is relatively small—about 25%—but of just the magnitude predicted by the model.

## Results

Previous attempts to define the role of Notch signalling in somite patterning have mostly relied on analysis of mutants, morphants and embryos in which specific genes have been overexpressed from the beginning of development. The final outcome then reflects the cumulative effect of the genetic disturbance and does not tell us whether a gene has acted early, or late, or both early and late in the history of a given somite [47].

In this paper we take a different approach, focusing on the zebrafish and blocking or overactivating the Notch signalling pathway abruptly part-way through the process of somitogenesis. To block, we use the gamma-secretase inhibitor DAPT [27,48]. To overactivate, we use heat shock in transgenic embryos to drive expression of NICD, the activated form of Notch [49,50].

If Notch signalling is required in the anterior part of the PSM, the effect on somite segmentation in either case should be more or less immediate, because the next somite to form consists of cells exiting from the anterior PSM. If Notch signalling is required only in the posterior part of the PSM, but is needed there to keep individual cells oscillating with adequate amplitude, the effect should be somewhat delayed, because cells leaving the posterior part of the PSM take five somite cycle times to traverse the anterior PSM before they form somites, and in this period disturbances of their *her1*/*her7* oscillations have no effect on their subsequent somitogenesis behaviour [28]. Lastly, if Notch signalling is needed only to maintain synchrony in the posterior PSM, the effect on somite formation should be seen only after a much longer delay, corresponding to the sum of the time taken for loss of synchrony in the posterior PSM and the time then taken by the desynchronized cells to traverse the anterior PSM. The time for desynchronization should be predictable from the number of somites that form normally in Notch pathway mutants or in embryos where Notch signalling has been blocked ab initio, if the formation of the first few somites is controlled in the same way as that of the subsequent ones.

### DAPT Treatment Results in Somite Defects with a Long Delay

**Figure 1:**
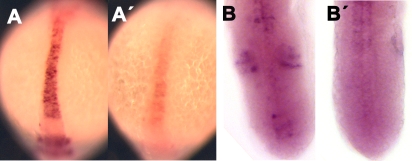
DAPT Treatment Blocks Notch Signalling Rapidly DAPT causes loss of *her4* expression (A) in the neural tube and (B) in the anterior PSM (where *her4* is normally expressed in a faint but detectable stripe). Wild-type embryos were treated with 100 μm DAPT or with DMSO (control) medium for 1 h and stained by ISH for *her4*. In each panel, the DMSO control is shown on the left, the DAPT-treated embryo on the right. Treatment was begun at the 13-somite stage for (A) and at the 15-somite stage for (B). For each case, at least 14 embryos were examined, and typical specimens were selected for this illustration.

To block Notch signalling in a temporally controlled manner, we immersed zebrafish embryos at somitogenesis stages in a DAPT solution [27,48]. To confirm the effectiveness of the block, we checked the expression of *her4*, which is a direct target of Notch signalling in the central nervous system and elsewhere [51–53]. After one hour of 100 μM DAPT treatment, *her4* expression was drastically reduced in comparison with control DMSO-treated embryos both in the neural tube and the anterior PSM (Figure 1), showing that the DAPT not only blocked Notch signalling, but also did this rapidly, within less than two ticks of the somite clock.

**Figure 2:**
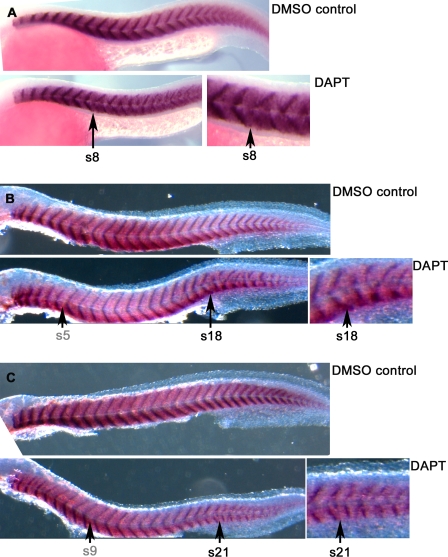
Blocking Notch Signalling Causes Somite Boundary Defects after a Long Delay Embryos were treated with 100 μm DAPT or with DMSO (control) medium and stained by ISH for *titin* at the end of somitogenesis to reveal somite boundaries. Treatment was begun (A) at 3 hpf, (B) at 5-somite stage, or (C) at 9-somite stage. Arrows with grey labels indicate stage at onset of DAPT treament; arrows with black labels indicate the level of the earliest defective somite. A detailed view of the region where disruption begins is shown to the right of each DAPT specimen.

Effects on somite boundary formation, however, were visible only after a long delay of about 6.5 hours: DAPT treatment of embryos at the 5- or 9-somite stage caused somite boundary defects at the level of 18.4 ± 1.1 (mean ± SD, *n* = 12) or 21.8 ± 1.9 (mean ± SD, *n* = 11) somites, respectively (Figure 2B and 2C). In other words, there was a delay of 13.4 ± 1.1 clock cycles before we saw defects in the first case, and of 12.8 ± 1.9 cycles in the second case. DAPT treatment starting much earlier, at 3hpf, 7 hours before the onset of somitogenesis, gave a phenotype like that of typical Notch pathway mutants, with boundary defects arising after 7.3 ± 1.3 (mean ± SD, *n* = 21) normal somites had formed (Figure 2A). Mara et al. [45], in a paper published just before the present work was submitted, have also recently examined the effects of DAPT treatment initiated at different stages in zebrafish; although their study has a different focus from our own, and they do not use their findings to draw the same conclusions, their data show a closely similar delayed effect of DAPT treatment on somite boundary formation.

One might wonder whether the delay in onset of somite defects could result from use of an inadequate dose of DAPT, giving only a partial blockade of Notch signalling. The 100 μM concentration used in our experiments described above was the highest possible, given the limit of solubility of DAPT. We did, however, examine batches of embryos exposed to lower doses of DAPT starting at 3 hpf. For 70 μM, 50 μM, and 35 μM, the onset of somite boundary defects was seen at 7.6 ± 1.0 (*n* = 24), 7.4 ± 1.0 (*n* = 24), and 7.6 ± 0.8 (*n* = 23) somites respectively—that is, at the same level as with our standard 100 μM dose. For a 20 μM dosage, onset was at 9.3 ± 2.4 (*n* = 26) somites, but with some embryos showing a defect on only one side. 10 μM doses gave no visible defect. We conclude that our standard 100 μM dose was enough to saturate the relevant targets of DAPT.

We also examined whether the duration of exposure to DAPT (at 100 μM) would affect the level of onset of somite defects. One hour of immersion in DAPT solution, starting at 7 hpf (i.e., three hours before appearance of the first somite), resulted in defects in 70% of the embryos, and half of these had defects only on one side; the level of onset in the embryos showing defects was at 10.7 ± 2.4 (*n* = 16) somites. (Note that the one hour of immersion of the embryo corresponds to a somewhat longer exposure of the cells to DAPT, if we take account of the wash-out delay.) Two hours of DAPT treatment resulted in defects in 90% of the embryos, of which 10% had defects only on one side; the level of onset was at 9.1 ± 2.0 (*n* = 20) somites. Three and four hours of DAPT treatment resulted in boundary defects in all of the embryos on both sides, with onset of defects at 8.2 ± 1.4 (*n* = 29) and 8.4 ± 1.2 (*n* = 26) somites, respectively. In all these DAPT pulse experiments, all of the embryos recovered normal segmentation posterior to the defect. Continuous exposure to the same dosage starting at the same time gave somite defects starting at 8.7 ± 1.3 (*n* = 24) somites, but without recovery. Evidently, when the duration of DAPT treatment is as short as one hour, the eventual loss of synchrony is often too slight to tip the tissue over the threshold for gross disruption of segmentation. The stochastic nature of the process means that one side of the embryo may be disrupted while the other is not; but in cases where such disruption is seen, even if only on one side of the embryo, the delay before onset of disruption is very nearly the same as when DAPT treatment is continuous. We conclude that the time of onset of somite disturbances is primarily a function of the time of onset of the DAPT blockade, and not of DAPT dosage or duration.

These findings all concur in showing, first of all, that Notch signalling is not required for the clock read-out process occurring in the anterior half of the PSM; for if it were required there, we should have seen somite segmentation defects within a few cycles after the onset of the Notch blockade. It seems that the boundary-formation hypothesis cannot be true for the zebrafish.

We can go further, and use these observations to judge the other two hypotheses. Our previous study [28] showed that disruption of oscillatory expression of the clock genes *her1*, *her7*, and *deltaC* during somitogenesis stages results in boundary defects with a delay of 5 somites, implying that the cells in the anterior half of the PSM are immune to the disruption of the segmentation clock, and that the segmentation pattern reflects the level at which the clock genes were expressed at the moment when the future somite cells were passing from the posterior into the anterior PSM region, 5 clock cycles before they segment overtly. From the delay we see following DAPT treatment, therefore, we can infer that the pattern of clock gene expression in the posterior PSM was not effectively disrupted until long after the beginning of DAPT treatment: for treatment beginning at the 5-somite stage, not until 13.4 ± 1.1 − 5, i.e., 8.4 ± 1.1, clock cycles had elapsed; for treatment beginning at the 9-somite stage, not until 12.8 ± 1.9 − 5, i.e., 7.8 ± 1.9, clock cycles had elapsed. From these delays, we should subtract the short period—2 clock cycles at most—required for DAPT to diffuse into the tissue and accomplish the block of Notch signalling. Thus we can say that, regardless of whether the DAPT treament begins at the 5-somite stage or the 9-somite stage, approximately 6 to 8 somite cycles must elapse from the time when the blockade of Notch signalling begins to the time when the pattern of clock gene expression is effectively disrupted.

For treatment beginning at 3hpf, 7 hours before the onset of somitogenesis, the calculation is slightly different. If we assume that the first somite is formed from cells in which the clock was set going synchronously by some shared developmental cue for the initiation of somitogenesis, we can infer that the critical oscillations were not significantly disrupted by the presence of Notch blockade until 7.3 ± 1.3 cycles after this initiating cue.

All these observations point to the same conclusion: Notch signalling cannot be directly required for genesis of the clock oscillations, since these evidently continue for 6 to 8 cycles after the onset of the blockade. For the zebrafish at least, the oscillation-generator hypothesis seems to be excluded.

**Figure 3:**
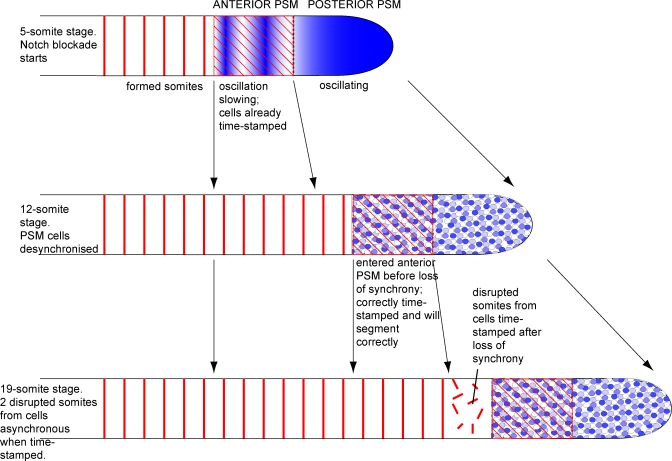
Cartoon to Show Why Somite Boundary Defects Appear only After a Long Delay Following Blockade of Notch Signaling

All the observations are, however, perfectly consistent with the synchronization hypothesis, if we assume that the cells in the posterior PSM continue oscillating when Notch signalling is blocked, but drift out of synchrony over the course of 6 to 8 cycles (Figure 3).

**Figure 4:**
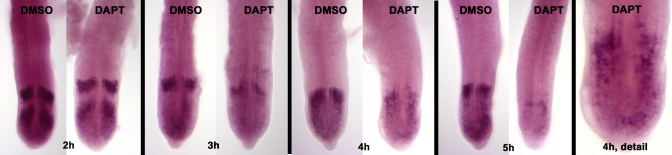
DAPT Treatment Disrupts Synchronized Oscillations of *her1* within 3–4 h Wild-type embryos were treated with 100 μm DAPT or with DMSO (control) medium and stained by ISH for *her1* for 2, 3, 4, or 5 h, as indicated. The rightmost panel is a detail of the 4-h specimen, showing the chaotic pattern of expression of *her1*. Treatment was begun at the 16-somite stage. For each case, at least 14 embryos were examined, and typical specimens were selected for this illustration.

To check this interpretation, we looked at the pattern of *her1* expression. This was normal after 1 or 2 hours of DAPT application (Figure 4). After 3 hours of DAPT treatment, corresponding to 6 clock cycles, the stripes in the anterior PSM, reflecting coordinated oscillations, were smeared into a quasi-uniform expression pattern in about 30% of embryos, though still distinguishable in the rest. After 4 and 5 hours, corresponding to 8 or 10 clock cycles, all embryos had lost all sign of organized stripiness in their expression pattern. Close examination showed that in such embryos the levels of expression varied chaotically from cell to cell, as expected if there was a loss of synchrony without a total failure of oscillation (see Figure 4). There also appeared to be some reduction in the mean level of expression; this was hard to quantify by in situ hybridization (ISH), but was measurable with the help of a reporter transgene, as we shall explain below.

Similar delayed disorganization of the stripe pattern was seen for *her7* and *deltaC* (unpublished data).

### NICD and the Her1/7 Proteins May Act Competitively on the *her1/7* Promoter to Maintain Synchronized Oscillations

Our DAPT data strongly support the hypothesis that Notch signalling in the PSM is needed to keep the cell clocks synchronized, and only for that. But how can Notch signalling do this? In a previous paper [29], we proposed a mathematical model that offered an answer. According to this model, the closely linked *her1* and/or *her7* genes are the pacemakers of a clock that operates in each PSM cell individually: their expression oscillates as a result of a delayed negative feedback loop, in which the Her1/7 protein products act back on the *her1/7* promoter to inhibit transcription. The oscillating levels of these proteins also drive oscillating expression of the Notch ligand DeltaC, and thereby activate Notch cyclically in the neighbouring cells. And activated Notch—that is, NICD—somehow combines with Her1/7 protein so as to modulate *her1/7* transcription in each cell and thereby entrain neighbours to the same rhythm.

Recent quantitative experiments have supported this mathematical model [28]. In computing the behaviour of the model [29], however, there was one detail where we made an assumption that is clearly wrong in the light of our present data. Although we allowed for different forms of combinatorial regulation of *her1*/*7* by Her1/7 in conjunction with NICD, we showed computations for the case where we assumed arbitrarily that the main regulation was multiplicative (i.e., described by multiplying an inhibitory term representing the effect of Her1/7 by a stimulatory term representing the effect of NICD). The assumption implied a zero level of expression of *her1/7* in the absence of Notch signalling, and the DAPT data show that this is wrong.

**Figure 5:**
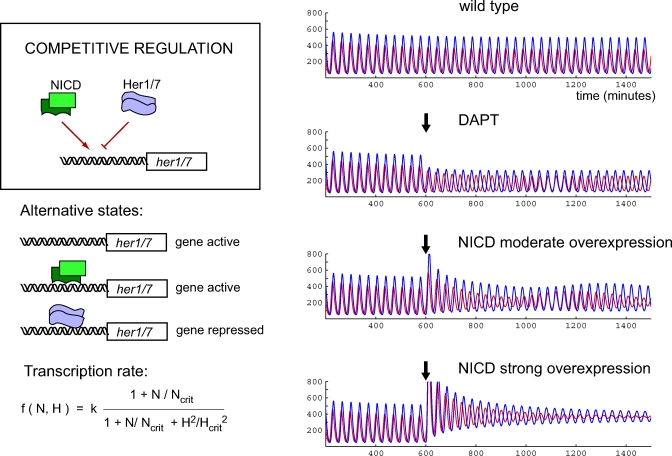
A Model of *her1/7* Gene Regulation by NICD and Her1/7 Protein, and the Predictions That It Makes The left column explains the basic hypothesis: NICD and Her1/7 protein compete to bind to a regulatory site of the *her1/7* gene. The function f (N,H) describes the resulting dependence of the rate of transcription of *her1/7* on the concentrations N and H of NICD and Her1/7 monomers respectively (see text and Text S1). The right column shows the predicted behaviour of the model when a perturbation is abruptly imposed at the time indicated by the arrow. The *red* and *blue* lines show the computed oscillating levels of expression of Her1 protein in two neighbouring cells whose free-running rhythms differ in period by 10%. In the “wild-type” condition, where Notch signalling operates normally, the two cells are entrained to oscillate in synchrony. In the “DAPT” condition, where Notch signalling is defective, synchrony is gradually lost. When a moderate uniform constitutive level of NICD (2 × N_crit_, see Text S1) is superimposed on the endogenous production, synchrony is again lost, and complex variations of expression amplitude occur in the long term. Finally, when a very high uniform constitutive level of NICD (20 × N_crit_) is superimposed on the endogenous production, synchrony is lost and the oscillations are severely damped.

We have therefore reconsidered how NICD and Her1/7 might jointly regulate *her1/7* transcription. One highly plausible possibility is that NICD and Her1/7 compete in some way to bind to the regulatory DNA of *her1/7*, as documented for some other gene regulatory proteins at other loci (e.g., [54]). For example, NICD and Her1/7 might bind at separate enhancers in the *her1/7* regulatory DNA, and these enhancers might then compete for binding at the promoter site (through DNA looping [55,56]). In this way, Her1/7 would inhibit *her1/7* transcription, but the presence of NICD would prevent it from doing so. A similar effect will be obtained if NICD and Her1/7 compete directly for binding to a regulatory site (Figure 5). We have recomputed the behaviour of the model on the assumption of competitive regulation, with the rule that when NICD is bound to the regulatory DNA, or neither NICD nor Her1/7 is bound, transcription is permitted; but when Her1/7 is bound, transcription is blocked. For simplicity, we assume that NICD and Her1/7 compete directly for a binding site. With this small correction of the original model (spelled out in detail in the Text S1), we reproduce all the essential properties described originally, plus the behaviours observed in the present work, including oscillations that are synchronized in the presence of normal Notch signalling but continue unsynchronized (though with reduced amplitude) when Notch signalling is either blocked or constitutive (Figure 5).

As always, however, the model fits the data only for appropriate choices of the parameter values. The parameters assumed in computing Figure 5 are the same as in [29], slightly updated according to our recent measurements of transcriptional delays and mRNA lifetimes [28], and with one additional adjustment: to match the present observations, we have to assign an appropriate strength to the influence of Delta-Notch signalling. This is represented in the model by the number of molecules of Delta required to drive NICD to the level at which it half-saturates its available binding sites. Figure 5 assumes for this parameter a value 0.4 times smaller than in the original computation [29].

### Loss of Notch Signalling Produces a Small Decrease in the Mean Level of Expression of a *her1* Reporter

We tested whether this new parameter choice is true to the experimental facts, by measuring how much the mean level of expression of the clock gene *her1* changes if Notch signalling is blocked. The model predicts a reduction by 25% (see Figure 5).

**Figure 6:**
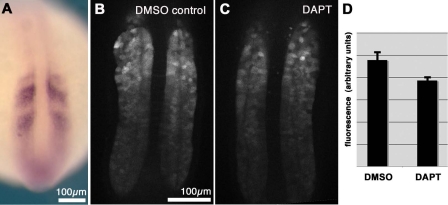
Effect of Loss of Notch Signalling on the Expression of a GFP Reporter for *her1* (A) ISH staining for *gfp* mRNA in a transgenic embryo shows a pattern of expression similar to the normal *her1* expression pattern. (B) GFP fluorescence of a transgenic embryo in the living state treated with DMSO (control), as seen by confocal microscopy in an optical section. Note that the magnification is higher than in (A), and the optical section shows only the PSM. (C) Corresponding embryo treated with DAPT and imaged in the same way as in (B). (D) Measured average fluorescence intensities in the PSM of transgenic embryos containing the reporter, after treatment with either 100 μM DAPT or DMSO (control) medium. Treatment was from 5.5 hpf up to 16-somite stage. Fluorescence levels were lower in the DAPT-treated embryos than in the controls by a factor of 0.81 ± 0.07 (mean ± SEM, *n* > 12 embryos of each type). Error bars show standard error of the mean.

For our measurements, we used a transgenic zebrafish line containing a reporter for *her1, her1:d1EGFP*, that allowed us to quantitate expression levels directly in vivo. The reporter transgene was based on a BAC (bacterial artificial chromosome) that comprised the whole of the *her1* and *her7* genomic region; it thus presumably included all the normal regulatory elements of *her1*. Into this BAC we inserted, by homologous recombination, the DNA for a destabilized form of GFP in the place of the *her1* coding sequence (see Materials and Methods). The 5′ and 3′ UTRs of *her1* were retained, so as to confer a short half-life on the GFP mRNA, which showed a typical cyclic expression pattern in the PSM of the transgenic fish (Figure 6A). The destabilized GFP protein encoded by this mRNA is reported (by Clontech) to have a half-life of one hour in cell cultures, and it appears to have a similar half-life in the zebrafish embryo. Thus it gives a visible fluorescence in the PSM, where *her1* is expressed, and lingers for a few somite cycles thereafter, so that it can also be seen in the two or three most recently formed somites. The relatively long lifetime of the GFP protein—about twice the length of a clock cycle—makes it difficult to detect temporal oscillation in the level of the GFP reporter in the posterior part of the PSM, but entails that the fluorescence observed serves as an indicator of the mean level of reporter gene expression, averaged over a clock cycle.

We examined batches of embryos from incrosses between parents both carrying the transgene but otherwise genetically normal. Using confocal microscopy, we compared the levels of the GFP reporter in living embryos treated from 50% epiboly to the 16-somite stage either with DAPT or with DMSO solution (corresponding to the vehicle in which DAPT was dissolved) as a control (Figure 6B–6D). After subtraction of background (estimated from control embryos that lacked the transgene), we found that levels of the reporter were lower in the PSM of DAPT-treated embryos than in the controls by a factor of 0.81 ± 0.07 (mean ± SEM, *n* > 12 embryos of each type). We conclude that Notch signalling is not absolutely required for expression of *her1* but does indeed modulate its expression (and presumably that of *her7* and other oscillating genes), altering the average level of protein product by roughly 20%, in good accordance with the theory.

### Heat-Shock–Triggered Overexpression of NICD also Disrupts Segmentation After a Long Delay

If Notch signalling is important only for maintenance of synchrony, we should expect that artificially imposed uniform steady activation of the Notch pathway should have much the same effect as a blockade. In both cases, the cells would be unable to use variations in clock-driven Notch signalling to display their clock cycle phase to their neighbours, and so would be unable to coordinate their clocks. Our mathematical model indeed shows this behaviour: when a steady level of “exogenous” Notch pathway activation is superimposed on the normal system, synchronization fails (Figure 5). The model also makes two other, less obvious, predictions, however. First, with moderately high levels of overexpression of NICD, the loss of synchrony goes with a *reduction* in the peak levels of *her1* and *her7* expression. Second, when NICD is very strongly overexpressed, the oscillations become damped and the system tends toward a state of uniform moderately high expression of *her1* and *her7*. Early disappearance of low minima of expression of these genes might be expected to lead to relatively early onset of somite boundary defects.

To test the effect of forced overactivation of the Notch pathway, we crossed fish containing an *hsp70:Gal4VP16* transgene (*Tg(hsp70I:Gal4vp16)vu22*) (a gift from B. Appel) with fish containing a *UAS:myc-notch1aICD* transgene (*Tg(UAS:myc-Notch1a-intra)kca3*) [49,50]. By heat-shocking the progeny, we could then drive production of the active intracellular domain of Notch1a, NICD, in those embryos that inherited both transgenes.

**Figure 7:**
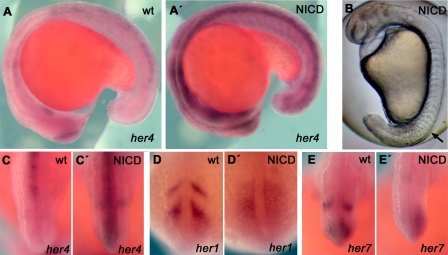
Overactivation of the Notch Pathway by NICD Causes Somite Boundary Defects After a Long Delay NICD overexpression was induced by 30-min heat shock in *hsp70:Gal4VP16*;*UAS:myc-notch1aICD* transgenic embryos. (A,A′) One hour after the end of a heat shock (delivered at the 16-somite stage), *her4* expression is strongly induced in the neural tube and other tissues, including the PSM (C,C′). (B) An embryo heat-shocked at 10 hpf, showing disturbances of somite boundary formation only after a long delay (as with DAPT treatment). The start of the boundary defects is marked by the *arrow*. (D,D′) Embryos heat-shocked at the five-somite stage, fixed 2.5 h later, and stained by ISH for *her1*. (E,E′) Embryos heat-shocked at the 16-somite stage, fixed 3 h later, and stained by ISH for *her7*. For both *her1* and *her7*, the stripy pattern seen in the controls is replaced by more uniform expression in the transgenic embryos, implying a breakdown of synchronized oscillation. Note that the levels of *her1* and *her7* expression in the transgenic embryos are lower than the peak levels in the normal controls. For each case in (A) to (E), at least 14 embryos were examined and typical specimens were selected for this illustration.

The onset of the disturbance of Notch signalling is rapid, driving upregulation of the target gene *her4* within 1.5 hours after the beginning of heat shock (Figure 7A′ and 7C′). As with DAPT, however, somite boundary defects were seen only after a much longer delay: a 30-minute heat shock at 10 hpf (the 0-somite stage), for example, generated boundary defects only after a delay of 15.7 ± 1.8 (mean ± SD, *n* = 10) somites (Figure 7B). Allowing for the delay required for production of NICD, this result is very similar to the one obtained with DAPT treatment. In batches of embryos heat-shocked at later (3- to 5-somite) stages, the delay before onset of visible defects was again long, but not so long, with defects becoming visible after 10 to 11 further somites had formed. (We do not know why the delay varies with the stage at the time of heat shock; one possibility is that there are variations in heat-shock efficacy, leading to differences in the induced level of NICD.) These findings are consistent with the predictions of our model for moderate to high overexpression of NICD (see Figure 5). Moreover, ISH analysis of the patterns of expression of *her1* and *her7* shows that the appearance of somite boundary defects is preceded by a disruption of the synchronized expression of these genes (see Figure 4), which tend toward an expression level that is uniform and lower than the peak seen in controls (Figure 7D–7E′), as the model predicts (see Figure 5). Thus all our observations appear to support the synchronization hypothesis, and are difficult to explain on the basis of the other hypotheses we have mentioned.

## Discussion

In this study, we have used timed perturbations of Notch signalling to discover at what step or steps it acts in the process of somite formation. This has allowed us to settle some questions that could not be answered by analysis of mutants and morphants, where Notch signalling is altered ab initio. We have found that blocking Notch signalling part way through somitogenesis disrupts the pattern of somite boundaries only after a delay, in the same way that a lack of Notch signalling from the very beginning of somitogenesis still allows the first few somites to form. The long delay before somite defects appear shows that Notch signalling plays no significant part in patterning of the prospective somite boundaries while the cells are in the anterior part of the PSM: its time of action must be earlier in the history of each somite, while the future somite cells are still in the posterior part of the PSM. Moreover, the length of the delay tells us that even within this population, the effects of loss of Notch signalling are not immediate, but take about 7 cycles of the somite clock to become significant.

### The Observations Support the Desynchronization Hypothesis

In our experiments, as in Notch pathway mutants and morphants, the eventual onset of abnormalities is correlated with a loss of uniformity in the levels of expression of oscillator genes in neighbouring PSM cells, suggesting that synchrony has been lost while individual cells have continued to oscillate. The most straightforward interpretation of our findings, therefore, is that the function of Notch signalling throughout normal somitogenesis is simply to maintain synchrony between adjacent cells in the posterior PSM, as proposed by Jiang et al. [15].

Loss of synchrony is presumably a manifestation of random variation in the free-running rhythm of the individual cells. One source of such variation lies in the stochastic nature of the association/dissociation reaction between Her1/7 protein and its DNA binding site. We have elsewhere shown theoretically that the strength of this source of noise is determined by the mean lifetime of the Her1/7-DNA bound state [29]. A value of about 1 minute for this parameter would give rise to random variation in the cycle time such that independently oscillating cells would take about 5 to 10 cycles to drift out of synchrony ([29] and calculations not shown), as observed. Whether this type of genetic noise is actually the main reason for loss of synchrony remains to be tested experimentally.

Whatever the source of cell-to-cell variability may be, to perform the task of maintaining synchrony, Notch signalling must exert some influence on the oscillators in the individual cells. We have used zebrafish containing a *her1* reporter transgene to measure the strength of this influence. By mathematical modelling, we have shown that the measured strength is entirely consistent with the synchronization hypothesis: normal Notch signalling at the observed level can maintain synchrony, while individual cells can still oscillate, though in an unsynchronized fashion, when Notch signalling is blocked or constitutively imposed.

### Notch Signalling Has No Demonstrable Function in Zebrafish Somite Segmentation beyond the Maintenance of Synchronized Oscillations in the Posterior PSM

Our results argue strongly against two ideas that have been proposed by other workers: that the function of Notch signalling is different for anterior and posterior somites [14,24,32,57,58], and that Notch signalling between cells in the anterior PSM is important in somite patterning [40–43]. Both these points need some commentary. While our findings indicate that there is no difference between anterior and posterior somites in the part played by Notch signalling, there is good evidence that the most anterior somites are special in some other aspects of their development. The distinctive developmental features of the anterior somites have been emphasized by Holley in a recent comprehensive review [14]. For example, they differ in their dependence on *her1* as opposed to *her7* [16,24], and the most anterior somite boundaries are particularly susceptible to disruption by loss of the *integrinalpha5* (*before eight*) gene [58,59]; cells destined to form anterior rather than posterior somites also express different members of the *tbx* gene family [60]. But this does not mean that the role of Notch signalling is different.

Our observations do not address the question of how oscillations start, at the point where PSM cells originate, although it has been suggested that Notch signalling may have a special role in this initiation process [45]. But the finding that the initial oscillations occur normally [46] and the first few somites form normally even when Notch signalling is blocked ab initio argues against the idea that Notch signalling is required for this initial step.

### Fish and Amniotes May Differ

We should emphasize that our findings relate only to the zebrafish. The somite patterning mechanisms in birds and mammals appear to be different in some significant ways [14,21]. In these species, Notch signalling may be essential to maintain the amplitude of oscillations in individual cells in the posterior PSM [34–36] and may have important functions in the anterior PSM [37–43].

There are also some other caveats. It is possible that our DAPT treatment did not block all Notch signalling absolutely, and that residual levels of Notch signalling were sufficient for function in the anterior PSM. Conversely, it is possible that our *hsp70:Gal4VP16*;*UAS:myc-notch1aICD* technique, by driving production of NICD to unphysiologically high levels, could have given a misleading impression as to the normal role of NICD. The concordance of our results from these two very different techniques, however, as well as the evidence from mutants and morphants, amounts to strong evidence that Notch signalling in zebrafish somitogenesis is responsible for synchronization of the cell oscillators and little else.

A further caveat is that our experiments only test the role of canonical Notch signalling—that is, Notch signalling that depends on γ-secretase and the production of NICD. In the fly wing disc, it has been suggested that Notch might play a part in boundary formation through a direct interaction with the actin cytoskeleton, independent of any activation of Notch by γ-secretase [61]. Our data do not exclude such a possibility in the context of somitogenesis, although it seems unlikely, given that mutations in *notch1a* (*deadly seven* mutations) cause practically the same disturbances of somite segmentation as does treatment with DAPT [2,45].

### The Oscillatory Behaviour of Individual Desynchronized Cells Remains To Be Analysed

One key aspect of our theory of the segmentation clock mechanism remains to be tested directly: we have not proved that individual cells continue to oscillate, rather than simply fluctuate randomly, in the absence of Notch signalling. Moreover, we have not established whether single cells continue to oscillate indefinitely in these conditions, or whether their oscillations are at first desynchronized and then eventually damped out. In the mouse, a luciferase reporter has been used to demonstrate the oscillations of individual cells in the PSM [62]; but in the zebrafish, with its much more rapid segmentation clock cycles, this feat has not yet been achieved. A conclusive analysis of dynamical phenomena such as the behaviour of the somite segmentation clock will require both time-resolved perturbations such as we have used in this paper, and real-time reporters that provide an accurate picture of the dynamics of gene expression in individual cells.

## Materials and Methods

### Fish stocks.

Fish were kept on a regular light-dark cycle at 28 °C. The *hsp70:Gal4VP16* line was a gift from Bruce Appel and the *UAS:myc-notch1aICD* line was a gift from Jose Campos-Ortega. *her1:d1EGFP* fish were generated as described below.

### Generation of *her1:d1EGFP* reporter transgene.

*her1:d1EGFP* DNA constructs were generated by a BAC recombination technique as described before [63]. CH211-283H6, which contains the complete *her1/7* locus plus adjacent sequence, was used as the host BAC. A sequence coding for a destabilized form of enhanced GFP, d1EGFP (Clontech), was inserted in place of the translated region of *her1*, preserving *her1* 5′ and 3′ UTRs. For purposes unrelated to the present paper, a long stretch of DNA (21kb) was inserted into the second intron of the *her7* gene, which reduced *her7* transcription drastically (100–200-fold; unpublished data). This residual expression of *her7* did not affect oscillatory gene expression and somite formation in the transgenic embryos (unpublished data).

Approximately 2 nl of a solution of the resulting BAC (15 μg/ml) was injected into freshly fertilized eggs together with I-SceI enzyme (New England Biolabs; 250 u/ml) and 0.5% phenol red in 1X I-SceI digestion buffer (New England Biolabs). Injected fish were raised to adulthood and screened for germ-line transmission by GFP fluorescence in the embryos that they spawned. One line was established showing GFP expression in the PSM. The transgene showed mendelian segregation (50% of the progeny of each fish inherited the transgene), implying that integration had occurred only at a single site in the genome.

### Fluorescence measurements and imaging.

Embryos were mounted in agar E3 solution. GFP fluorescence measurements were made from optical sections obtained with a Perkin-Elmer spinning-disk confocal microscope with 20/0.75W Nikon objective. Nontransgenic embryos were used as a control for background fluorescence. Images were analysed using ImageJ software. Background fluorescence was subtracted before the statistical calculations.

Contrast and brightness in the figures shown were linearly adjusted using Photoshop.

### Heat-shock procedures.

Embryos were kept at 28 °C until the desired stage for heat shock. They were then transferred to pre-warmed E3 medium in a 37 °C incubator for 30 minutes, and returned to 28 °C for further development.

### DAPT treatment and scoring.

DAPT from Calbiochem (#565770) was made up as an 8mM stock solution in DMSO; this was diluted to 100 μM with E3 medium for use. Embryos, with chorions torn but not completely removed, were transferred into the diluted DAPT or control medium containing the same concentration of DMSO. Embryos were incubated in this at 28 °C until the time of fixation. They were then fixed immediately in ice-cold buffered 4% formaldehyde for ISH. The position of boundary defects in each embryo was determined by averaging the positions of defects on the left and right sides of that embryo. This average number was then used to calculate the population average for the onset of boundary defects. In scoring the positions of defects, we had to take account of the fact that loss of synchrony, and the corresponding disruption of somite segmentation, are not instantaneous all-or-none events. There was, however, a relatively abrupt threshold effect, such that posterior to a certain level (definable with an accuracy of about one somite width), segmentation became grossly disordered, in the sense that the somite boundaries posterior to this level, as marked by *titin* expression, appeared fragmented and randomly positioned. We took this as our criterion for the onset of a segmentation defect. In some cases, as would be expected, slight alterations in the shaping of somite boundaries preceded the onset of the gross disruption.

### In situ hybridization.

In situ hybridization was performed according to standard protocols. Digoxigenin-labelled RNA probes were as previously described for *her1* [51], *her4* [52], *her7* [16,24], and *deltaC* [64]. The *titin* probe corresponded to 1467 nucleotides from the 8^th^ exon of the titin gene. The *gfp* probe corresponded to nucleotides 1–733 of the coding sequence of the Venus GFP variant.

### Mathematical modelling.

We used the model of Lewis [29], modified to describe competitive interaction between NICD and Her1/7 protein at the regulatory site of *her1* and *her7*, and with minor parameter changes, as explained in the text and in Figure 5. Details of the computation, in the form of a Mathematica (version 5.2) notebook, are given as Text S1.

## Supporting Information

Text S1 A Mathematical Model of the Somitogenesis Oscillator and the Role of Notch Signalling in Coupling Adjacent Cells This annotated Mathematica notebook describes two coupled oscillatory cells and computes their behaviour in the deterministic (no-noise) case, exploring the predicted effects of blocking or overactivating the Notch signalling pathway. The model is that of Lewis [29], modified to describe competitive interaction between NICD and Her1/7 protein at the regulatory site of *her1* and *her7*, and with minor parameter changes, as explained in the text and in Figure 5 of the main article.
(2.9 MB PDF)
