# Supplementary material for: Notch Signalling Synchronizes the Zebrafish Segmentation Clock but Is Not Needed To Create Somite Boundaries
Source: PLoS Genet. 2008 Feb 1;4(2):e15. doi: 10.1371/journal.pgen.0040015 (PMC2222926; doi:10.1371/journal.pgen.0040015)
Supplement: Supplementary file 1 [file pgen.0040015.sd001.pdf]

Two coupled oscillatory cells: numerical simulation of the deterministic (no-noise) case, with competitive interaction between regulatory molecules at the *her1/7* promoter

Assumptions

■ What's new

This model is the same as that published in Lewis (2003), with the following modifications:
1. We assume that the transcriptional delays and mRNA lifetimes have values based on the measurements of Giudicelli et al. (2007) (with "initiation delay" = 4 minutes, and the lifetimes assumed to be 40% shorter than the measured upper limits and thus approximately the same as in Lewis (2003)).
2. We assume that the critical concentration of Delta is 0.4 times lower than in the previous calculation
3. Most importantly, we assume that NICD (activated Notch, generated in proportion to the amount of Delta in the neighbouring cell) interacts with the Her1/7 protein complex competitively to regulate transcription of *her7*. The details of this simple competitive interaction are spelled out below.
4. To see the consequences of abruptly switching on a blockade of Notch activation (DAPT treatment), or of switching on a steady level of constitutive NICD production (heat-shock experiment), we allow the system to evolve according to the standard "wild-type" parameters up to a certain time point, and then switch on the perturbation.

Note that although we adhere to the original formulation in which Her1 and Her7 are assumed to combine as a heterodimer to inhibit transcription, the behaviour is practically the same if we assume that the inhibition is exerted by Her1 or Her7 acting as a homodimer (simulations not shown).

■ Description of the system - two cells interacting via Delta-Notch signalling and each containing a Her1/7 oscillator

In each of the two cells, the quantities of protein and message for her1, her7 and delta obey the following equations, in which, for clarity, we have omitted the index distinguishing values in one cell from those in the other

$$\frac{d\,p_{\text{her1}}}{dt} = [\,a_{\text{her1}}\; m_{\text{her1}}\,]_{t-T_{p_{\text{her1}}}} - b_{\text{her1}}\; p_{\text{her1}}(t)$$
$$\frac{d\,p_{\text{her7}}}{dt} = [a_{\text{her7}}\; m_{\text{her1}}\,]_{t-T_{p_{\text{her7}}}} - b_{\text{her7}}\; p_{\text{her7}}(t)$$
$$\frac{d\,p_{\text{delta}}}{dt} = [\,a_{\text{delta}}\; m_{\text{delta}}\,]_{t-T_{p_{\text{delta}}}} - b_{\text{delta}}\; p_{\text{delta}}(t)$$
$$\frac{d\,m_{\text{her1}}}{dt} = [f_{\text{her1}}\;(p_{\text{her1}},\; p_{\text{her7}},\; \tilde{p}_{\text{delta}})]_{t-T_{m_{\text{her1}}}} - c_{\text{her1}}\; m_{\text{her1}}(t)$$
$$\frac{d\,m_{\text{her7}}}{dt} = [f_{\text{her7}}\;(p_{\text{her1}},\; p_{\text{her7}},\; \tilde{p}_{\text{delta}})]_{t-T_{m_{\text{her7}}}} - c_{\text{her7}}\; m_{\text{her7}}(t)$$
$$\frac{d\,m_{\text{delta}}}{dt} = [f_{\text{delta}}\;(p_{\text{her1}},\; p_{\text{her7}},\; \tilde{p}_{\text{delta}})]_{t-T_{m_{\text{delta}}}} - c_{\text{delta}}\; m_{\text{delta}}(t)$$

Here, $[\,....]_{t-T}$ denotes the value of the expression in the square brackets evaluated at the timepoint $t-T$,
that is, with delay T, and $\tilde{p}$ denotes the value of p in the neighbouring cell;

$$f_{\text{her1}}\;(p_{\text{her1}},\; p_{\text{her7}},\; \tilde{p}_{\text{delta}}) =$$
$$k_{\text{her1}}\left(r_0 + r_d\,\frac{\tilde{\phi}_d}{1+\tilde{\phi}_d} + r_h\,\frac{1}{1+\phi_{\text{her1}}\,\phi_{\text{her7}}} + r_{\text{hd}}\,\frac{1+\tilde{\phi}_d}{1+\tilde{\phi}_d+\phi_{\text{her1}}\,\phi_{\text{her7}}}\right)$$
$$f_{\text{her7}}\;(p_{\text{her1}},\; p_{\text{her7}},\; \tilde{p}_{\text{delta}}) =$$
$$k_{\text{her7}}\left(r_0 + r_d\,\frac{\tilde{\phi}_d}{1+\tilde{\phi}_d} + r_h\,\frac{1}{1+\phi_{\text{her1}}\,\phi_{\text{her7}}} + r_{\text{hd}}\,\frac{1+\tilde{\phi}_d}{1+\tilde{\phi}_d+\phi_{\text{her1}}\,\phi_{\text{her7}}}\right)$$
$$f_{\text{delta}}\;(p_{\text{her1}},\; p_{\text{her7}},\; \tilde{p}_{\text{delta}}) =$$
$$k_{\text{delta}}\left(s_0 + s_d\,\frac{\tilde{\phi}_d}{1+\tilde{\phi}_d} + s_h\,\frac{1}{1+\phi_{\text{her1}}\,\phi_{\text{her7}}} + s_{\text{hd}}\,\frac{1+\tilde{\phi}_d}{1+\tilde{\phi}_d+\phi_{\text{her1}}\,\phi_{\text{her7}}}\right)$$

where ϕ denotes the reduced value of each protein concentration p, that is, its absolute value divided by the critical value p_0

$$\phi_d = p_d / p_{0\,d}\,,\quad \phi_{\text{her1}} = p_{\text{her1}} / p_{0\,\text{her}}\,,\quad \phi_{\text{her7}} = p_{\text{her7}} / p_{0\,\text{her}}$$

and the parameters r_0, r_d, r_h, and r_{hd}, assumed to add up to 1,
represent the relative importance of unregulated transcription,
transcription regulated (positively) by Delta / Notch activity alone, transcription regulated (negatively) by Her1 / 7 alone,
and transcription regulated combinatorially by Delta / Notch activity (positively) and Her1 / 7 (negatively).

■ Competitive regulation

The form of the combinatorial regulation (the r_{hd} or s_{hd} term) is derived as follows:
Let n and q denote the concentrations of NICD and of the Her1/7 protein complex in the cell, and let g_0, g_n, and g_q be respectively the probabilities that the regulatory DNA of *her1/7* has neither of these proteins bound to it, has NICD bound, or has Her1/7 bound. We assume that binding of NICD and of Her1/7 is competitive, so that the two molecules cannot bind simultaneously, and that when NICD is bound to the regulatory DNA, or neither NICD nor Her1/7 is bound, transcription is permitted; but when Her1/7 is bound, transcription is blocked. Then

$g_0 + g_n + g_q = 1$,
$g_0\, n = n_{crit}\, g_n$,
$g_0\, q = q_{crit}\, g_q$,
and
$g_{active} = g_0 + g_n$,

where n_{crit} and q_{crit} are dissociation constants, and g_{active} is the probability that *her1/7* is open for transcription. Solving these equations, we get

$$g_{active} = \frac{q_{crit}\,(n_{crit} + n)}{q_{crit}\, n + n_{crit}\,(q_{crit} + q)} = \frac{1 + n'}{1 + n' + q'}$$

where $n' = n / n_{crit}$ and $q' = q / q_{crit}$. We assume that n is directly proportional to the amount of Delta protein in the neighbouring cell, so that $n' = n / n_{crit} = \tilde{p}_d / p_{0\,d} = \tilde{\phi}_d$ (if we define $p_{0\,d}$ appropriately). q, the concentration of the dimeric DNA-binding complex of Her1/7, is assumed proportional to the product of the individual Her1 and Her7 protein concentrations, so that $q' = q / q_{crit} = (p_{her1} / p_{0\,her})(p_{her7} / p_{0\,her}) = \phi_{her1}\, \phi_{her7}$ (if we define $p_{0\,her}$ appropriately). This gives the formulae specified above for the wild-type system; and we calculate its behaviour, as before, for the case where $r_0 = r_d = r_h = s_0 = s_d = s_{hd} = 0$, so that the functions f reduce to:

$$f_{her1}\,(p_{her1},\ p_{her7},\ \tilde{p}_{delta}) = k_{her1}\, \frac{1 + \tilde{\phi}_d}{1 + \tilde{\phi}_d + \phi_{her1}\, \phi_{her7}}$$

$$f_{her7}\,(p_{her1},\ p_{her7},\ \tilde{p}_{delta}) = k_{her7}\, \frac{1 + \tilde{\phi}_d}{1 + \tilde{\phi}_d + \phi_{her1}\, \phi_{her7}}$$

$$f_{delta}\,(p_{her1},\ p_{her7},\ \tilde{p}_{delta}) = k_{delta}\, \frac{1}{1 + \phi_{her1}\, \phi_{her7}}$$

For a system in which Notch activation is artificially blocked, the equations become:

$$f_{her1}\,(p_{her1},\ p_{her7},\ \tilde{p}_{delta}) = k_{her1}\, \frac{1}{1 + \phi_{her1}\, \phi_{her7}}$$

$$f_{her7}\,(p_{her1},\ p_{her7},\ \tilde{p}_{delta}) = k_{her7}\, \frac{1}{1 + \phi_{her1}\, \phi_{her7}}$$

$$f_{delta}\,(p_{her1},\ p_{her7},\ \tilde{p}_{delta}) = k_{delta}\, \frac{1}{1 + \phi_{her1}\, \phi_{her7}}$$

For a system in which exogenous NICD is artificially added, at a concentration that is ν-fold higher than the normal critical concentration, the equations become:

$$f_{\text{her1}}\,(p_{\text{her1}},\; p_{\text{her7}},\; \tilde{p}_{\text{delta}}) = k_{\text{her1}}\;\frac{1+\tilde{\phi}_d+\nu}{1+\tilde{\phi}_d+\nu+\phi_{\text{her1}}\,\phi_{\text{her7}}}$$

$$f_{\text{her7}}\,(p_{\text{her1}},\; p_{\text{her7}},\; \tilde{p}_{\text{delta}}) = k_{\text{her7}}\;\frac{1+\tilde{\phi}_d+\nu}{1+\tilde{\phi}_d+\nu+\phi_{\text{her1}}\,\phi_{\text{her7}}}$$

$$f_{\text{delta}}\,(p_{\text{her1}},\; p_{\text{her7}},\; \tilde{p}_{\text{delta}}) = k_{\text{delta}}\;\frac{1}{1+\phi_{\text{her1}}\,\phi_{\text{her7}}}$$

Computation

NOTATION

```
(*
  Notation:
    x[[n,i]] denotes value of x at time-step n in cell i, where x may be a protein concentration or an mRNA concentration.
    Protein concentrations have initial letter p, mRNA concentrations initial letter m. Subsequent letters distinguish specific proteins and mRNA species - h1 for her1, h7 for her7, d for Delta.
    Thus ph7[[100,2]] is the concentration of Her7 protein in cell #2 at time-step 100; mh7[[100,2]] is the mRNA concentration of her7 in the same cell and time.
    Kinetic parameters, which may differ from cell to cell but are assumed independent of time, are denoted by initial letters as follows (with subsequent letters as above to identify the molecule referred to):
    a - protein synthesis rate per mRNA molecule
    b - protein degradation rate
    c - mRNA degradation rate
    delay - delay from initiation (the controlled step) to completion of synthesis of active form of molecule (for Delta protein, this will include time for transport to cell surface and for activation of Notch)
    crit - critical number of molecules of given protein per cell, i.e. number at which it exerts half-maximal regulatory effect
    f - function relating transcription initiation rate to protein concentrations
    k - transcription initiation rate constant per diploid gene pair (in specification of function f)
    eps - elementary time step used in computation
    Thus, for example, bh1[[1]] is the degradation rate for Her1 protein in cell #1
    Mostly, kinetic parameters are taken to be the same for both cells, so that they take the form parameter = value*{1,1}
  *)
```

DEFAULT PARAMETERS

```
(*  GENERAL CASE : HER1 AND HER7 DIFFERENT,
 CELLS DIFFERENT IN FREE-RUNNING RHYTHM, COUPLED VIA NOTCH SIGNALLING;
 DEFAULT PARAMETERS ARE GIVEN HERE -
```

```
  SUBSEQUENT PROGRAM BLOCKS MAY OVERRIDE THESE*)
minute = 1;
 eps = 0.3;
 tfinal = 600;
  (*  time step and duration of simulation  *)
 ah1 = ah7 = ad = 4.5 * {1, 1} / minute;
 (*  protein synthesis rates per mRNA molecule for Her1,
  Her7 & Delta, for the two cells  *)
 bh1 = bh7 = bd = 0.23 * {1, 1} / minute;
 (*  protein degradation rates,
  i.e. inverse lifetimes for Her1, Her7 & Delta  *)
 ch1 = (1 / (6.6 * 0.6)) * {1, 1} / minute;
 ch7 = (1 / (8.1 * 0.6)) * {1, 1} / minute;
 cd = (1 / (6.1 * 0.6)) * {1, 1} / minute;
 (*  mRNA degradation rates,i.e. inverse lifetimes for her1,
  her7 & delta, from Giudicelli et al. (2007),assuming that true
   lifetimes are 40 % less than the estimated upper limits   *)
 delayinit = 4 minute;
  (* Initiation delay, Tinit, as defined in Giudicelli et al. (2007)*)
 delaymh1 = (3.8 + delayinit) * {.95, 1.05} minute;
  (*  time to make a molecule of her1 mRNA, from Giudicelli et al. (2007) *)
 delayph1 = 2.8 * {1, 1} minute;
  (*  time to make a molecule of Her1 protein *)
 delaymh7 = (3.7 + delayinit) * {0.95, 1.05} minute;
  (*  time to make a molecule of her7 mRNA, from Giudicelli et al. (2007) *)
 delayph7 = 1.7 * {1, 1} minute;
  (*  time to make a molecule of Her7 protein *)
 delaymd = (8.4 + delayinit) * {1, 1} minute;
  (*  time to make a molecule of delta mRNA, from Giudicelli et al. (2007) *)
 delaypd = 20.5 * {1, 1} minute;
  (*  time to make a molecule of Delta protein
   (mature, delivered to the cell surface, activating Notch. *)

 kh1 = 33 / minute;
  (* maximal synthesis rate of her1 mRNA   *)
 kh7 = 33 / minute;
  (* maximal synthesis rate of her7 mRNA   *)
 kd = 33 / minute;
  (*  maximal rate of synthesis of delta mRNA  *)

 critph = 40 ;
  (*  critical number of molecules of Her1/Her7
   protein per cell for inhibition of transcription  *)
 critpd = 400;
  (*  critical number of molecules of Delta protein
    per cell for activation of Notch. MODIFIED - WAS 1000 *)

 r0 = 0;
  (*  fraction of her1/7 transcription that is unregulated   *)
 rh = 0;
  (* fraction of her1/7 transcription
```

```
  that is regulated by Her1/Her7 alone  *)
rd = 0;
 (* fraction of her1/7 transcription that is regulated by Delta alone   *)
rhd := 1 - r0 - rh - rd;
 (* fraction of her1/7 transcription that is
  regulated combinatorially by Her1/Her7 and Delta  *)

s0 = 0;
 (*  fraction of delta transcription that is unregulated   *)
sh = 1;
 (* fraction of delta transcription
  that is regulated by Her1/Her7 alone  *)
sd = 0;
 (* fraction of delta transcription that is regulated by Delta alone   *)
shd := 1 - s0 - sh - sd;
 (* fraction of delta transcription that is
  regulated combinatorially by Her1/Her7 and Delta  *)
unblocked = 1; (*takes value 1 in the normal case,
 0 in embryos treated with DAPT to block Notch activation*)
exonicd = 0; (* takes value 0 in the normal case,
 or a value >>1 in embryos where NICD is artificially overexpressed*)

fr[x1_, x7_, y_] :=
 r0 + rd * ((y + exonicd) / (1 + (y + exonicd))) + rh * 1 / (1 + x1 * x7) +
  rhd * ((1 + unblocked * y + exonicd) / (1 + (unblocked * y + exonicd) + x1 * x7));
fs[x1_, x7_, y_] := s0 + sd * y / (1 + y) + sh * 1 / (1 + x1 * x7) +
   shd * (y / (1 + y)) (1 / (1 + x1 * x7));
 (* rate of transcription initiation, for her1/7 and delta respectively,
 relative to max possible rate,
 expressed as function of reduced protein concentrations,
 i.e. of concentrations divided by their critical values. MODIFIED fr *)

fh1[xh1_, xh7_, yd_] := kh1 * fr[xh1 / critph, xh7 / critph, yd / critpd];
fh7[xh1_, xh7_, yd_] := kh7 * fr[xh1 / critph, xh7 / critph, yd / critpd];
fd[xh1_, xh7_, yd_] := kd * fs[xh1 / critph, xh7 / critph, yd / critpd];
 (*  absolute rates of transcription initiation,
 in transcripts per minute per cell, for her1,
 her7 and delta as functions of numbers of protein molecules per cell*)

nbrs[{x_, y_}] = {y, x};
 (*  If {x,y}[[i]] is the value of a variable in cell #i,
 nbrs[{x,y}][[i]] is its value in the neighbour (s) of cell #i *)
```

```
Null^33
```

SETTINGS FOR SPECIFIC CASE, OVERRIDING DEFAULTS

```
(*  SPECIAL CASES: SET PARAMETERS HERE TO OVERRIDE DEFAULTS, E.G.
     INDEPENDENT CELLS (no Notch communication)   - SET rd=rdh=0;
     HER1 AND HER7 IDENTICAL
    (EQUIVALENT TO DIMERIC REGULATION BY HER1 ALONE) - SET delaymh1=
  delaymh7, delayph1=delayph7, etc.    *)
(* Default settings except as follows and in the list
  of 'variants' of the outermost Do loop just below *)

tfinal = 1500;
tstartperturb = 600;
```

INITIAL CONDITION

```
ph1 = ph7 = mh1 = mh7 = pd = md = Table[{0, 0}, {t, 0, tfinal, eps}];
 (* initial condition - all concentrations zero  *)
```

COMPUTATION AND DISPLAY OF RESULTS

■ Set parameters

```
baselinedelaypd = 20 * {1, 1};
variants = {1};
setparams := (
  delaypd = baselinedelaypd * variants[[nv]];

  ndelaymh1 = IntegerPart[delaymh1 / eps];
  ndelayph1 = IntegerPart[delayph1 / eps];
  ndelaymh7 = IntegerPart[delaymh7 / eps];
  ndelayph7 = IntegerPart[delayph7 / eps];
  ndelaypd = IntegerPart[delaypd / eps];
  ndelaymd = IntegerPart[delaymd / eps];
  nstartperturb = IntegerPart[tstartperturb / eps];
  nfinal = IntegerPart[tfinal / eps];
  r0 = 0; rd = 0; rh = 0;
 )
```

■ Time development algorithm

```
calculatedevelopment := (
  j = Which[i == 1, 2, i == 2, 1];
  nph1 = ndelayph1[[i]];
  nph7 = ndelayph7[[i]];
  npd = ndelaypd[[i]];
  nmh1 = ndelaymh1[[i]];
  nmh7 = ndelaymh7[[i]];
  nmd = ndelaymd[[i]];
  ph1[[n, i]] = ph1[[n - 1, i]] +
    eps (If[n > nph1, ah1[[i]] * mh1[[n - nph1, i]], 0] - bh1[[i]] * ph1[[n - 1, i]]);
  ph7[[n, i]] = ph7[[n - 1, i]] +
    eps (If[n > nph7, ah7[[i]] * mh7[[n - nph7, i]], 0] - bh7[[i]] * ph7[[n - 1, i]]);
  pd[[n, i]] = pd[[n - 1, i]] +
    eps (If[n > npd, ad[[i]] * md[[n - npd, i]], 0] - bd[[i]] * pd[[n - 1, i]]);
  mh1[[n, i]] = mh1[[n - 1, i]] +
    eps (If[n > nmh1, fh1[ph7[[n - nmh1, i]], ph7[[n - nmh1, i]], pd[[n - nmh1, j]]],
        fh1[0, 0, 0]] - ch1[[i]] * mh1[[n - 1, i]]);
  mh7[[n, i]] = mh7[[n - 1, i]] + eps (If[n > nmh7,
        fh7[ph7[[n - nmh7, i]], ph7[[n - nmh7, i]], pd[[n - nmh7, j]]],
        fh7[0, 0, 0]] - ch7[[i]] * mh7[[n - 1, i]]);
  md[[n, i]] = md[[n - 1, i]] + eps (If[n > nmd, fd[ph7[[n - nmd, i]], ph7[[n - nmd,
           i]], pd[[n - nmd, j]]], fd[0, 0, 0]] - cd[[i]] * md[[n - 1, i]]);
 )
```

■ Output display algorithm

```
<< Graphics`MultipleListPlot`
outputdisplay := (
pth1 = Transpose[Table[{n * eps, ph1[[n, i]]}, {n, 1, nfinal}, {i, 1, 2}]];
  pth7 = Transpose[Table[{n * eps, ph7[[n, i]]}, {n, 1, nfinal}, {i, 1, 2}]];
ptd = Transpose[Table[{n * eps, pd[[n, i]]}, {n, 1, nfinal}, {i, 1, 2}]];
  mth1 = Transpose[Table[{n * eps, mh1[[n, i]]}, {n, 1, nfinal}, {i, 1, 2}]];
mth7 = Transpose[Table[{n * eps, mh7[[n, i]]}, {n, 1, nfinal}, {i, 1, 2}]];
  mtd = Transpose[Table[{n * eps, md[[n, i]]}, {n, 1, nfinal}, {i, 1, 2}]];

  Print[StyleForm[SequenceForm[
      "   ah1 = ", ah1,
      "   ah7 = ", ah7,
      "   ad = ", ad,
      "\n   bh1 = ", bh1,
      "   bh7 = ", bh7,
      "   bd = ", bd,
      "\n   ch1 = ", ch1,
      "   ch7 = ", ch7,
      "   cd = ", cd,
      "\n   kh1 = ", kh1 ,
      "   kh7 = ", kh7 ,
      "   kd = ", kd ,
      "\n   critph = ", critph,
      "   critpd = ", critpd,
      "\n Fractional contributions to regulation of her1/7:   r0 = ", r0,
      "   rd = ", rd,
      "   rh = ", rh,
      "   rhd = ", rhd,
      "\n Fractional contributions to regulation of delta:   s0 = ", s0,
      "   sd = ", sd,
      "   sh = ", sh,
      "   shd = ", shd,
      "\n   exonicd = ", exonicd,
      "   unblocked = ", unblocked,
      "\n   delaymh1 = ", delaymh1,
      "   delaymh7 = ", delaymh7,
      "   delaymd = ", delaymd,
      "\n   delayph1 = ", delayph1,
      "   delayph7 = ", delayph7,
      "   delaypd = ", delaypd
     ], FontSize → 10]]
Print[StyleForm[SequenceForm[
      "\n   Her1 delay in cells 1 and 2 = ", delayph1 + delaymh1,
      "      Her7 delay in cells 1 and 2 = ", delayph7 + delaymh7,
      "\n   DeltaC delay in cells 1 and 2 = ", delaypd + delaymd
     ], FontSize → 10]]

   (*  Work out period, amplitude, damping factor *)
```

```
    Do[
     (Do[
       If[mh1[[n + 1, i]] < mh1[[n, i]] && mh1[[n, i]] > mh1[[n - 1, i]],
        (tmaxpenult = tmaxlast; tmaxlast = n * eps;
         mmaxpenult = mmaxlast; mmaxlast = mh1[[n, i]])],
       {n, 2, nfinal - 1}];
      Do[
       If[mh1[[n + 1, i]] > mh1[[n, i]] && mh1[[n, i]] < mh1[[n - 1, i]],
        (tminpenult = tminlast; tminlast = n * eps;
         mminpenult = mminlast; mminlast = mh1[[n, i]])],
       {n, 2, nfinal - 1}];
      Print["CELL # ", i, ":   period = ", tmaxlast - tmaxpenult];
      Print["her1 mRNA amplitude = ",
       mmaxlast - mminlast, "     amplitude decrement factor = ",
       (mmaxlast - mminlast) / (mmaxpenult - mminpenult)]),
     {i, 1, 2}];

tstartplot = 200;
  MultipleListPlot[mth1[[1]], mth1[[2]], PlotJoined → {True, True} ,
    PlotStyle → {RGBColor[1, 0, 0], RGBColor[0, 0, 1]},
    SymbolShape → {None, None}, PlotRange → {{tstartplot, tfinal}, {0, 100}},
    AxesOrigin → {tstartplot, 0} , AspectRatio → 0.2,
    ImageSize → 72 * 8, PlotLabel → "\n her1 mRNA"];
MultipleListPlot[mth7[[1]], mth7[[2]], PlotJoined → {True, True} ,
    PlotStyle → {RGBColor[1, 0, 0], RGBColor[0, 0, 1]},
    SymbolShape → {None, None}, PlotRange → {{tstartplot, tfinal}, {0, 50}},
    AspectRatio → 0.2, ImageSize → 72 * 8, PlotLabel → "\n her7 mRNA"];
(* MultipleListPlot[mtd[[1]],mtd[[2]], PlotJoined→{True, True} ,
      PlotStyle→{RGBColor[1,0,0],RGBColor[0,0,1]},
      SymbolShape→{None,None}, PlotRange→{{tstartplot,tfinal},{0,50}},
      AspectRatio→0.2, ImageSize→72*8, PlotLabel→"\n deltaC mRNA"]; *)
grHer1Protein = MultipleListPlot[pth1[[1]], pth1[[2]],
     PlotJoined → {True, True} , PlotStyle →
      {RGBColor[1, 0, 0], RGBColor[0, 0, 1]}, SymbolShape → {None, None},
     PlotRange → {{tstartplot, tfinal}, {0, 20 * critph}}, AspectRatio → 0.2,
     ImageSize → 72 * 8, PlotLabel → "\n Her1 protein"];
MultipleListPlot[pth7[[1]], pth7[[2]], PlotJoined → {True, True} ,
    PlotStyle → {RGBColor[1, 0, 0], RGBColor[0, 0, 1]}, SymbolShape →
     {None, None}, PlotRange → {{tstartplot, tfinal}, {0, 20 * critph}},
    AspectRatio → 0.2, ImageSize → 72 * 8, PlotLabel → "\n Her7 protein"];
(*MultipleListPlot[ptd[[1]],ptd[[2]], PlotJoined→{True, True} ,
      PlotStyle→{RGBColor[1,0,0],RGBColor[0,0,1]}, SymbolShape→
       {None,None}, PlotRange→{{tstartplot,tfinal},{0,2*critpd}},
      AspectRatio→0.2, ImageSize→72*8, PlotLabel→"\n DeltaC protein"];*)
 )
```

- **Do it!**

```
Do[
  (
   setparams;
   exonicd = 0; unblocked = 1;
   Do[calculatedevelopment, {n, 2, nstartperturb}, {i, 1, 2}];
  exonicd = 0; unblocked = 0;
  Do[calculatedevelopment,
    {n, nstartperturb + 1, nfinal}, {i, 1, 2}]; outputdisplay;
  ),
  {nv, 1, Length[variants]}
 ];
Display["grHer1Protein1.eps", grHer1Protein, "EPS"];
```

```
   ah1 = {4.5, 4.5}   ah7 = {4.5, 4.5}   ad = {4.5, 4.5}
   bh1 = {0.23, 0.23}   bh7 = {0.23, 0.23}   bd = {0.23, 0.23}
   ch1 = {0.252525, 0.252525}   ch7 =
 {0.205761, 0.205761}   cd = {0.273224, 0.273224}
   kh1 = 33   kh7 = 33   kd = 33
   critph = 40   critpd = 400
 Fractional contributions to regulation of her1/7:   r0 =
 0   rd = 0   rh = 0   rhd = 1
 Fractional contributions to regulation of delta:   s0 =
 0   sd = 0   sh = 1   shd = 0
   exonicd = 0   unblocked = 0
   delaymh1 = {7.41, 8.19}   delaymh7 =
 {7.315, 8.085}   delaymd = {12.4, 12.4}
   delayph1 = {2.8, 2.8}   delayph7 = {1.7, 1.7}   delaypd = {20, 20}

   Her1 delay in cells 1 and 2 = {10.21, 10.99}
      Her7 delay in cells 1 and 2 = {9.015, 9.785}
   DeltaC delay in cells 1 and 2 = {32.4, 32.4}

CELL # 1:   period = 31.8

her1 mRNA amplitude = 12.1631     amplitude decrement factor = 0.999677

CELL # 2:   period = 33.6

her1 mRNA amplitude = 17.5589     amplitude decrement factor = 1.00018
```

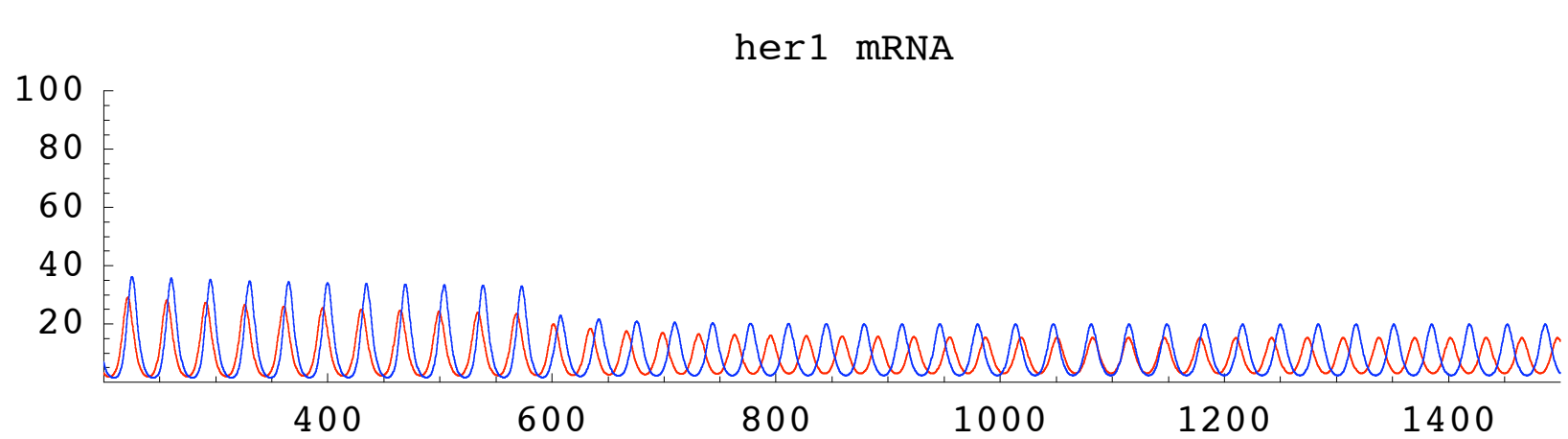

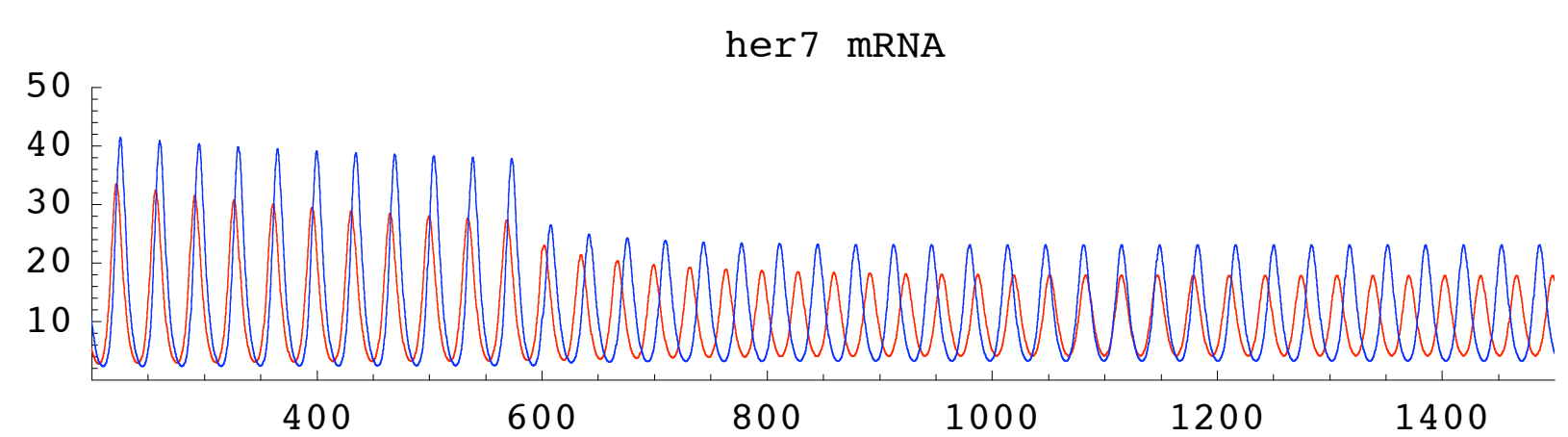

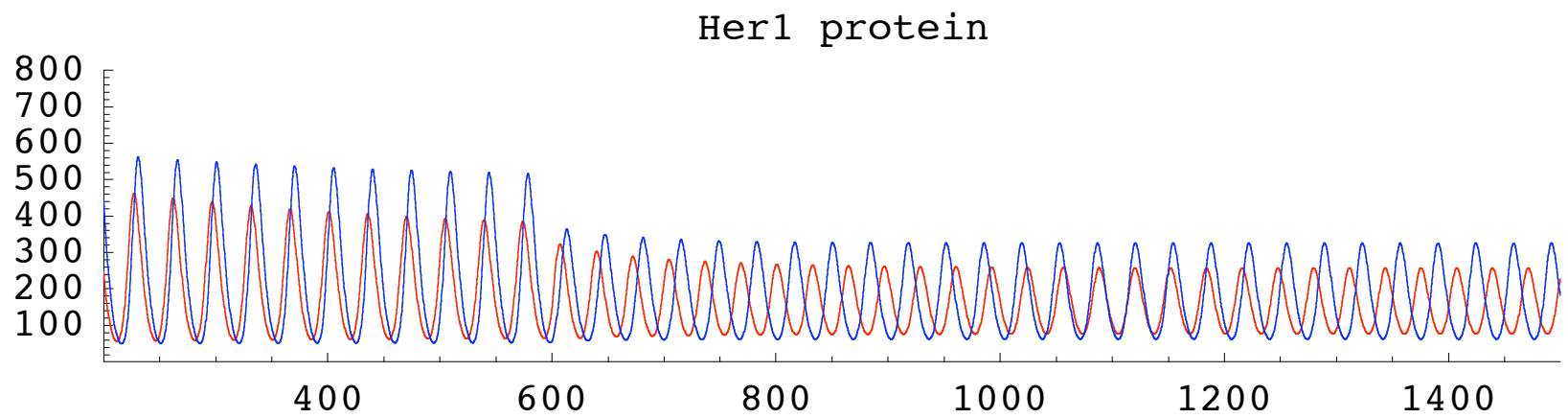

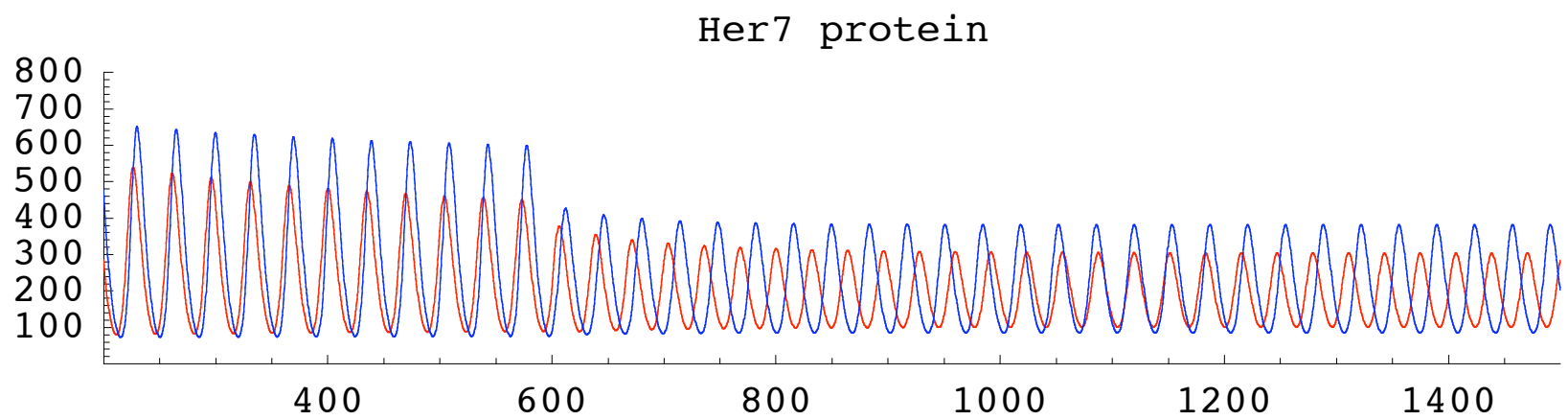


```
mean[xlist_, nstart_, nstop_] :=
  Plus @@ Take[xlist, {nstart, nstop}] / (nstop - nstart + 1);
Print["mean her1 protein in cells #1 and #2 before perturbation = ",
  mean[ph1, IntegerPart[nstartperturb / 2], nstartperturb]];
Print["mean her1 protein in cells #1 and #2 after perturbation = ",
  mean[ph1, IntegerPart[nstartperturb * 1.5], nstartperturb * 2]];
```

```
mean her1 protein in cells #1 and #2 before perturbation = {198.194, 238.417}

mean her1 protein in cells #1 and #2 after perturbation = {155.929, 173.849}
```

■ Do it!

```
Do[
   (
    setparams;
    exonicd = 0; unblocked = 1;
    Do[calculatedevelopment, {n, 2, nstartperturb}, {i, 1, 2}];
   exonicd = 2; unblocked = 1;
   Do[calculatedevelopment,
     {n, nstartperturb + 1, nfinal}, {i, 1, 2}]; outputdisplay;
   ),
   {nv, 1, Length[variants]}
  ];
Display["grHer1Protein2.eps", grHer1Protein, "EPS"];
```

```
  ah1 = {4.5, 4.5}   ah7 = {4.5, 4.5}   ad = {4.5, 4.5}
  bh1 = {0.23, 0.23}   bh7 = {0.23, 0.23}   bd = {0.23, 0.23}
  ch1 = {0.252525, 0.252525}   ch7 =
{0.205761, 0.205761}   cd = {0.273224, 0.273224}
  kh1 = 33   kh7 = 33   kd = 33
  critph = 40   critpd = 400
Fractional contributions to regulation of her1/7:   r0 =
0   rd = 0   rh = 0   rhd = 1
Fractional contributions to regulation of delta:   s0 =
0   sd = 0   sh = 1   shd = 0
  exonicd = 2   unblocked = 1
  delaymh1 = {7.41, 8.19}   delaymh7 =
{7.315, 8.085}   delaymd = {12.4, 12.4}
  delayph1 = {2.8, 2.8}   delayph7 = {1.7, 1.7}   delaypd = {20, 20}

  Her1 delay in cells 1 and 2 = {10.21, 10.99}
     Her7 delay in cells 1 and 2 = {9.015, 9.785}
  DeltaC delay in cells 1 and 2 = {32.4, 32.4}

CELL # 1:   period = 30.3

her1 mRNA amplitude = 6.32045     amplitude decrement factor = 0.902434

CELL # 2:   period = 33.3

her1 mRNA amplitude = 20.2359     amplitude decrement factor = 0.969414
```

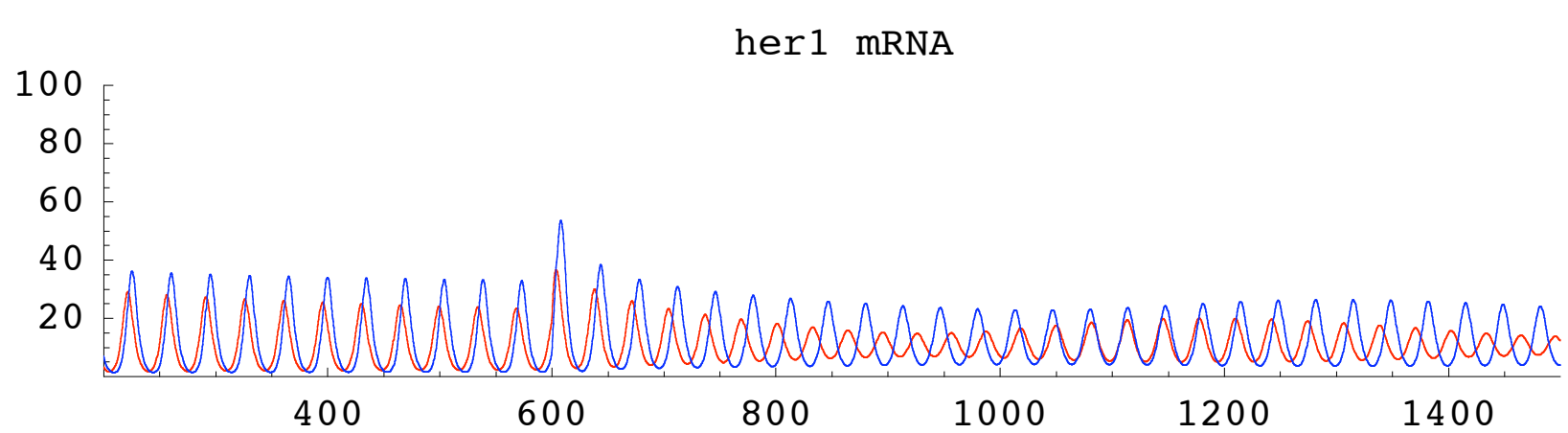

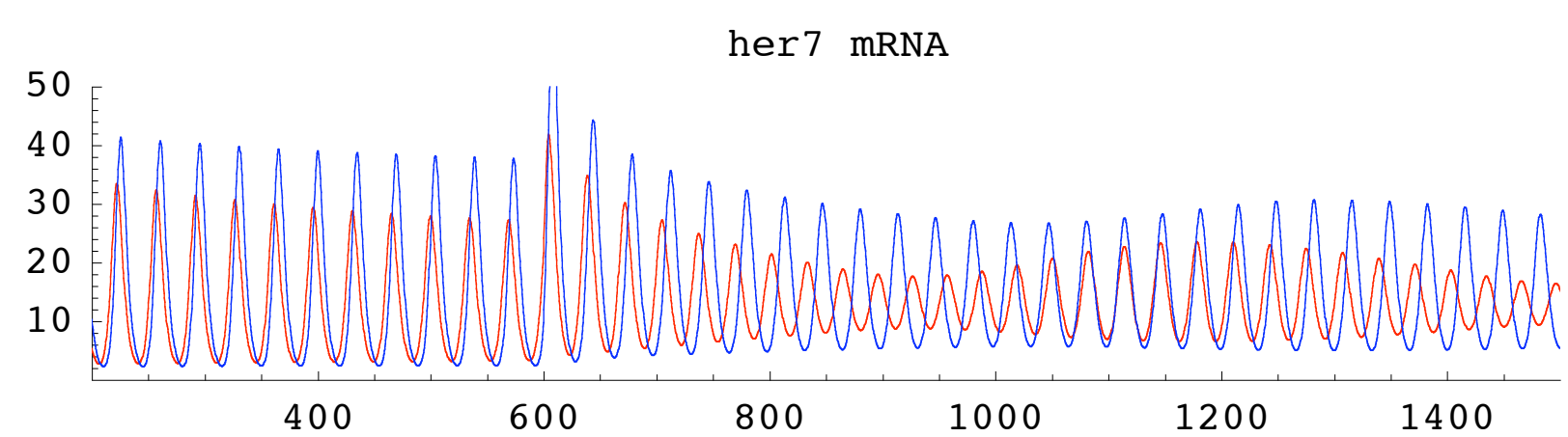

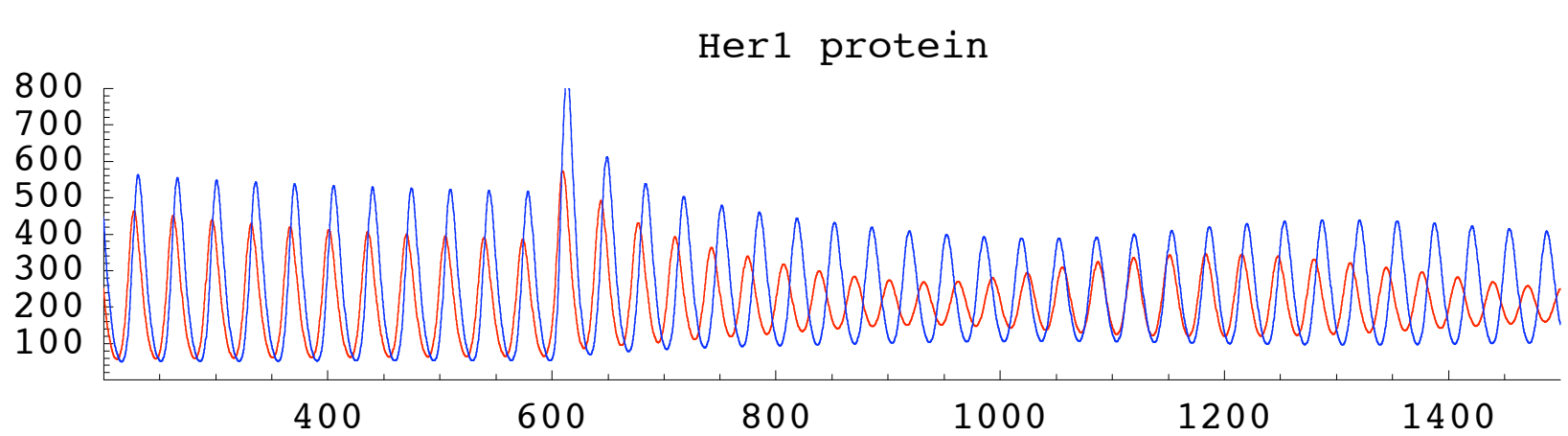

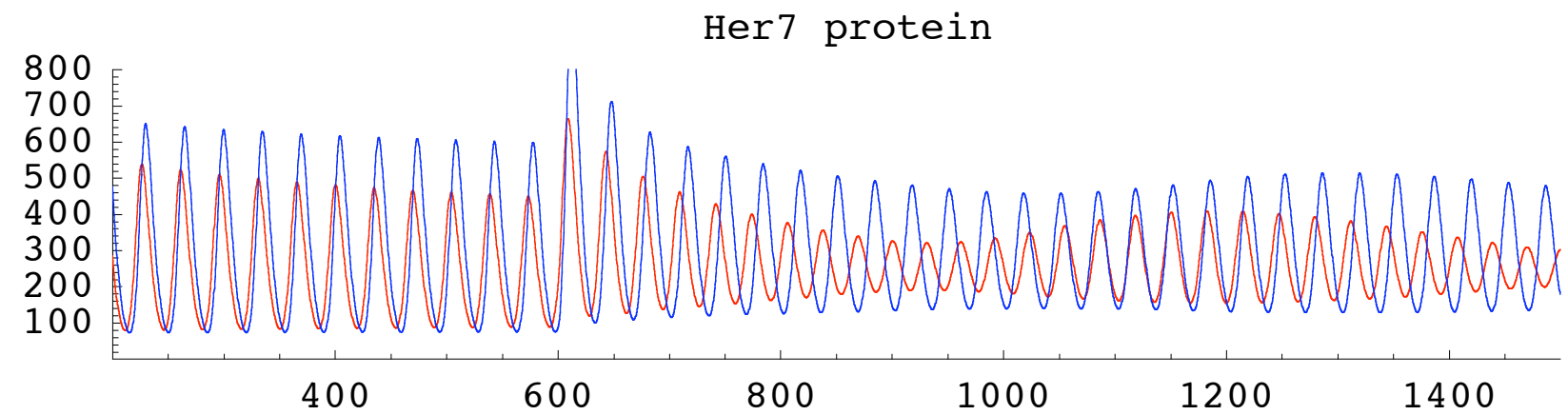


```
mean[xlist_, nstart_, nstop_] :=
  Plus @@ Take[xlist, {nstart, nstop}] / (nstop - nstart + 1);
Print["mean her1 protein in cells #1 and #2 before perturbation = ",
  mean[ph1, IntegerPart[nstartperturb / 2], nstartperturb]];
Print["mean her1 protein in cells #1 and #2 after perturbation = ",
  mean[ph1, IntegerPart[nstartperturb * 1.5], nstartperturb * 2]];
```

```
mean her1 protein in cells #1 and #2 before perturbation = {198.194, 238.417}

mean her1 protein in cells #1 and #2 after perturbation = {213.25, 232.643}
```

■ Do it!

```
Do[
  (
   setparams;
   exonicd = 0; unblocked = 1;
   Do[calculatedevelopment, {n, 2, nstartperturb}, {i, 1, 2}];
  exonicd = 0; unblocked = 1;
  Do[calculatedevelopment,
    {n, nstartperturb + 1, nfinal}, {i, 1, 2}]; outputdisplay;
  ),
  {nv, 1, Length[variants]}
 ];
Display["grHer1Protein0.eps", grHer1Protein, "EPS"];
```

```
   ah1 = {4.5, 4.5}   ah7 = {4.5, 4.5}   ad = {4.5, 4.5}
   bh1 = {0.23, 0.23}   bh7 = {0.23, 0.23}   bd = {0.23, 0.23}
   ch1 = {0.252525, 0.252525}   ch7 =
 {0.205761, 0.205761}   cd = {0.273224, 0.273224}
   kh1 = 33   kh7 = 33   kd = 33
   critph = 40   critpd = 400
 Fractional contributions to regulation of her1/7:   r0 =
 0   rd = 0   rh = 0   rhd = 1
 Fractional contributions to regulation of delta:   s0 =
 0   sd = 0   sh = 1   shd = 0
   exonicd = 0   unblocked = 1
   delaymh1 = {7.41, 8.19}   delaymh7 =
 {7.315, 8.085}   delaymd = {12.4, 12.4}
   delayph1 = {2.8, 2.8}   delayph7 = {1.7, 1.7}   delaypd = {20, 20}
```

```
Her1 delay in cells 1 and 2 = {10.21, 10.99}
   Her7 delay in cells 1 and 2 = {9.015, 9.785}
DeltaC delay in cells 1 and 2 = {32.4, 32.4}

CELL # 1:    period = 34.5

her1 mRNA amplitude = 18.8479      amplitude decrement factor = 0.999401

CELL # 2:    period = 34.5

her1 mRNA amplitude = 29.9582      amplitude decrement factor = 0.999397
```

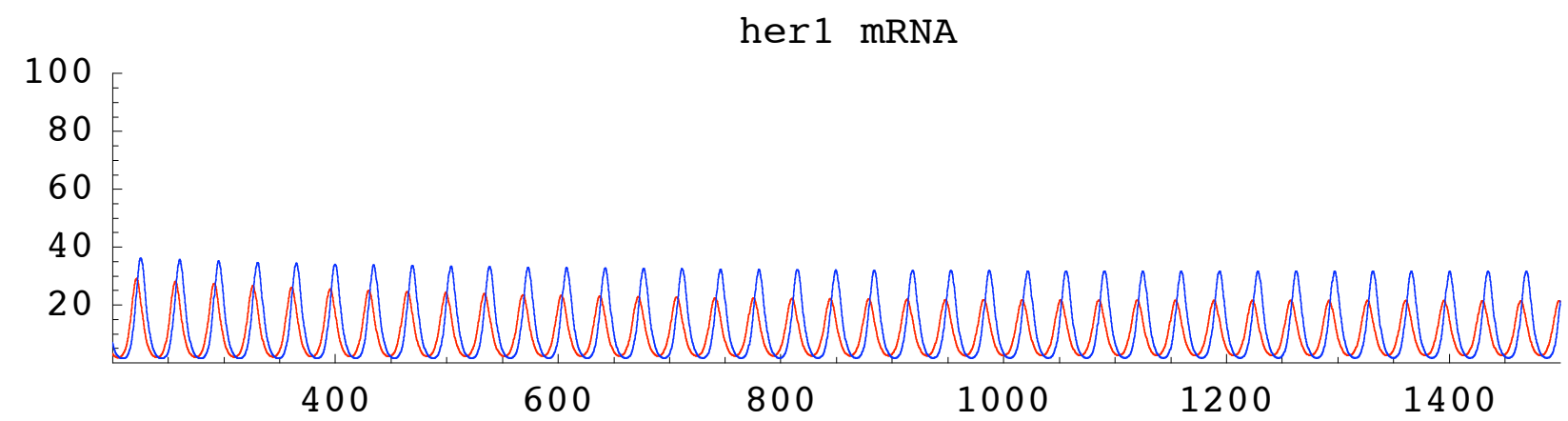

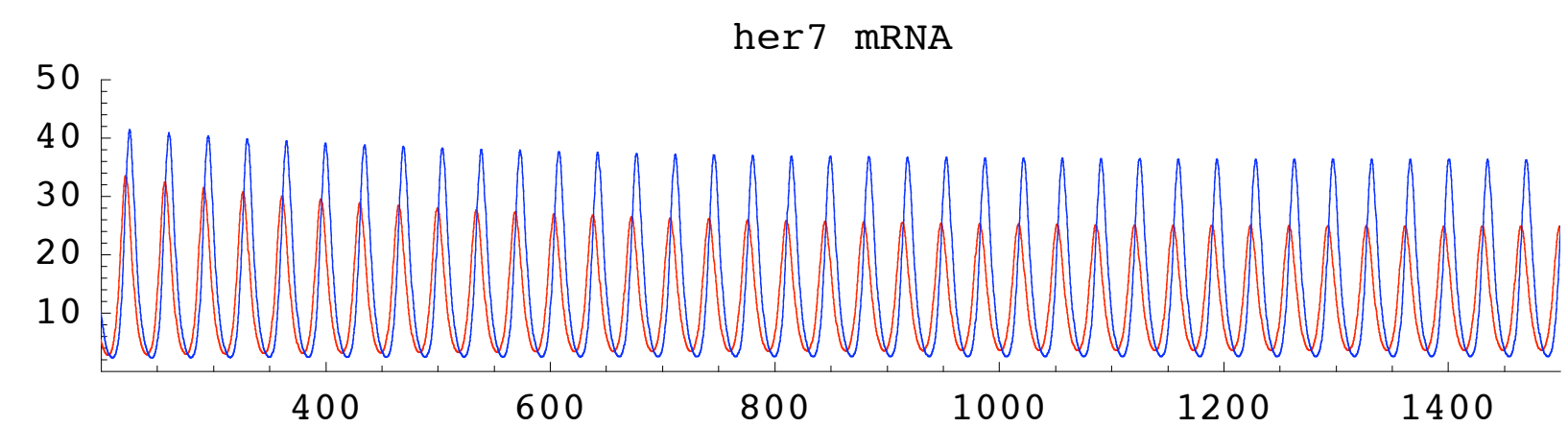

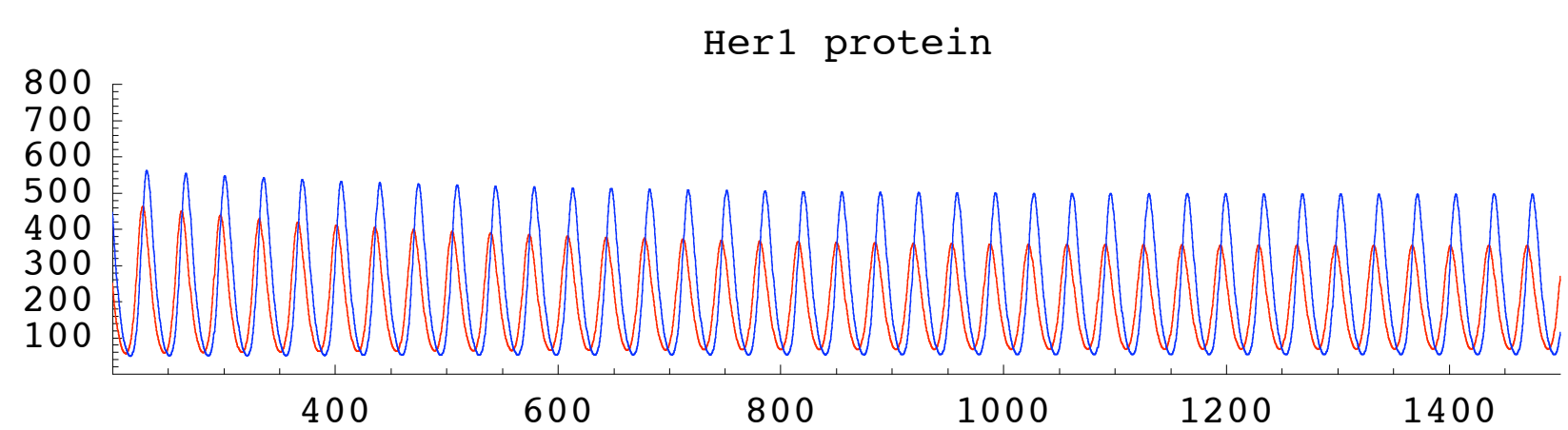

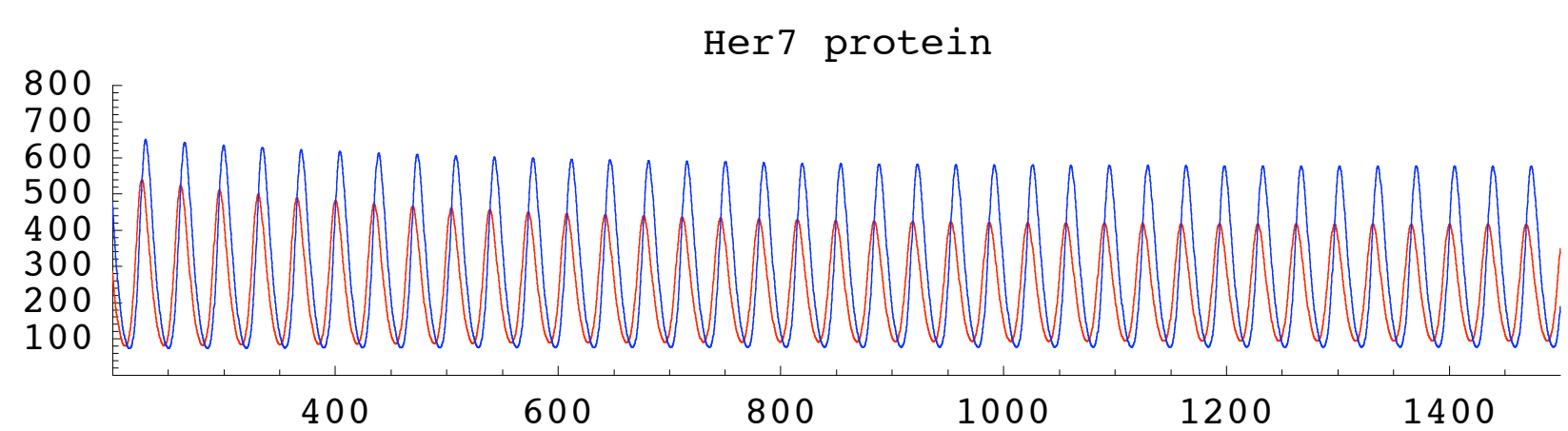

```
Do[
  (
   setparams;
   exonicd = 0; unblocked = 1;
   Do[calculatedevelopment, {n, 2, nstartperturb}, {i, 1, 2}];
  exonicd = 20; unblocked = 1;
  Do[calculatedevelopment,
     {n, nstartperturb + 1, nfinal}, {i, 1, 2}]; outputdisplay;
  ),
  {nv, 1, Length[variants]}
 ];
Display["grHer1Protein3.eps", grHer1Protein, "EPS"];
```

```
   ah1 = {4.5, 4.5}   ah7 = {4.5, 4.5}   ad = {4.5, 4.5}
   bh1 = {0.23, 0.23}   bh7 = {0.23, 0.23}   bd = {0.23, 0.23}
   ch1 = {0.252525, 0.252525}   ch7 =
 {0.205761, 0.205761}   cd = {0.273224, 0.273224}
   kh1 = 33   kh7 = 33   kd = 33
   critph = 40   critpd = 400
 Fractional contributions to regulation of her1/7:   r0 =
 0   rd = 0   rh = 0   rhd = 1
 Fractional contributions to regulation of delta:   s0 =
 0   sd = 0   sh = 1   shd = 0
   exonicd = 20   unblocked = 1
   delaymh1 = {7.41, 8.19}   delaymh7 =
 {7.315, 8.085}   delaymd = {12.4, 12.4}
   delayph1 = {2.8, 2.8}   delayph7 = {1.7, 1.7}   delaypd = {20, 20}

   Her1 delay in cells 1 and 2 = {10.21, 10.99}
      Her7 delay in cells 1 and 2 = {9.015, 9.785}
   DeltaC delay in cells 1 and 2 = {32.4, 32.4}
```

```
CELL # 1:   period = 31.5

her1 mRNA amplitude = 1.45974     amplitude decrement factor = 0.896109

CELL # 2:   period = 32.7

her1 mRNA amplitude = 8.26608     amplitude decrement factor = 0.970033
```

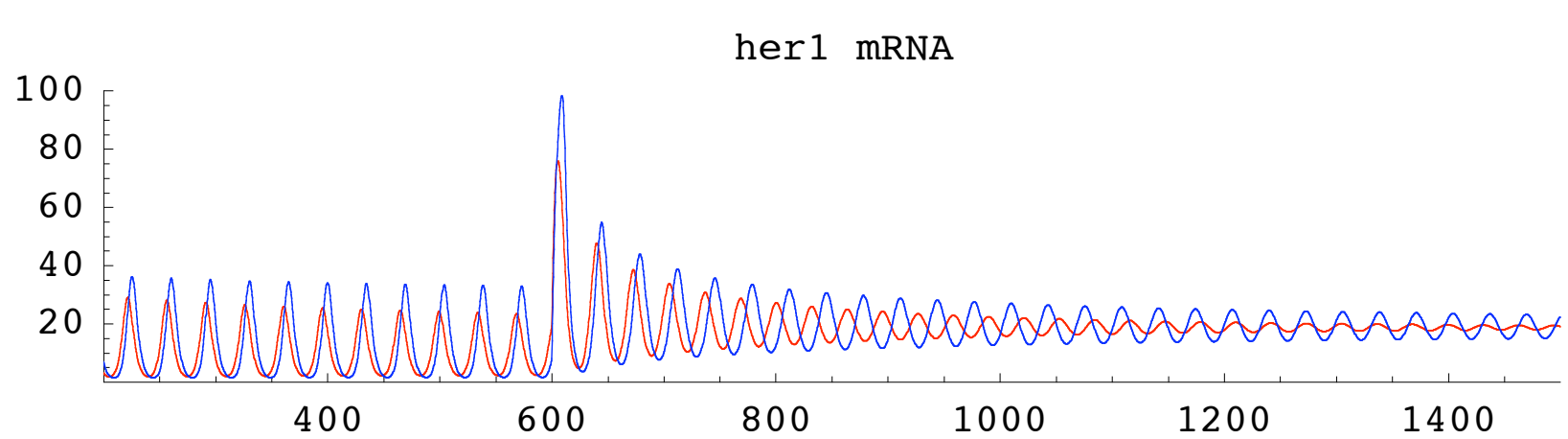

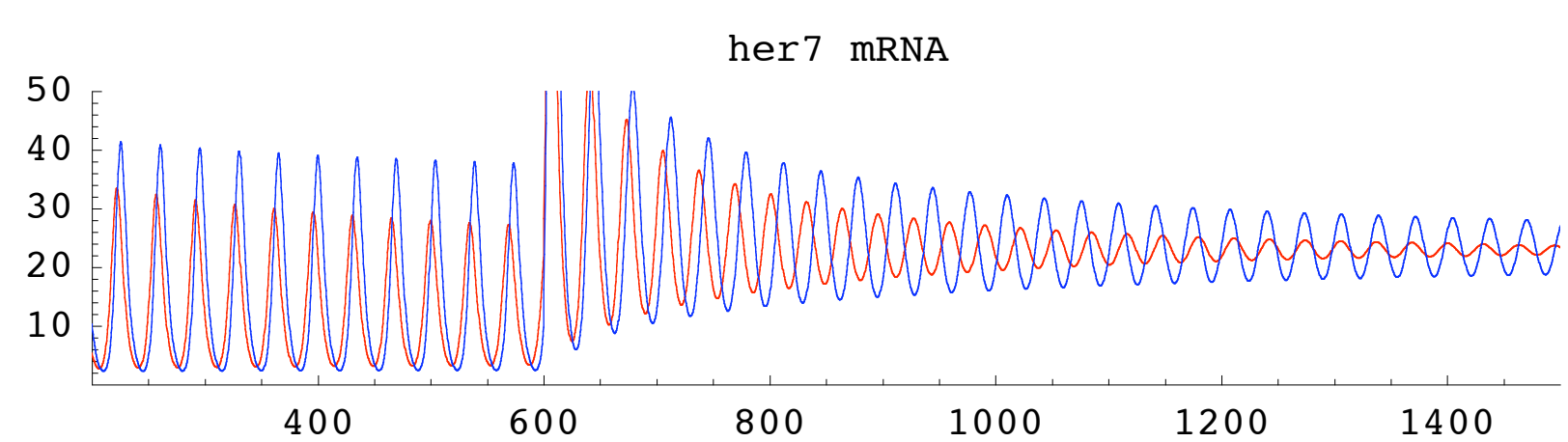

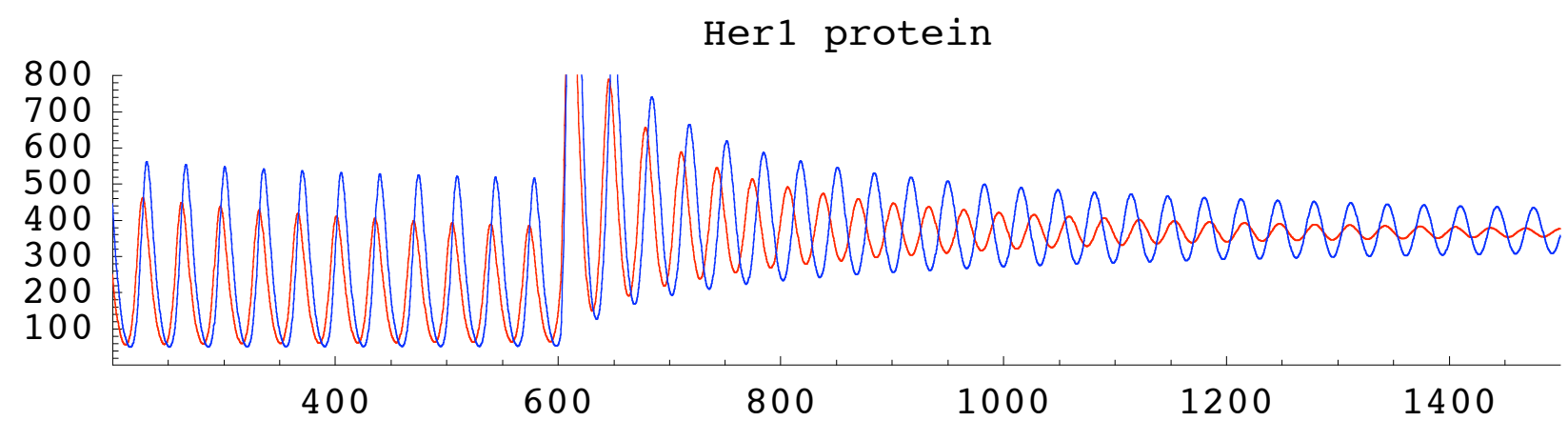
Her1 protein
800
700
600
500
400
300
200
100
400
600
800
1000
1200
1400

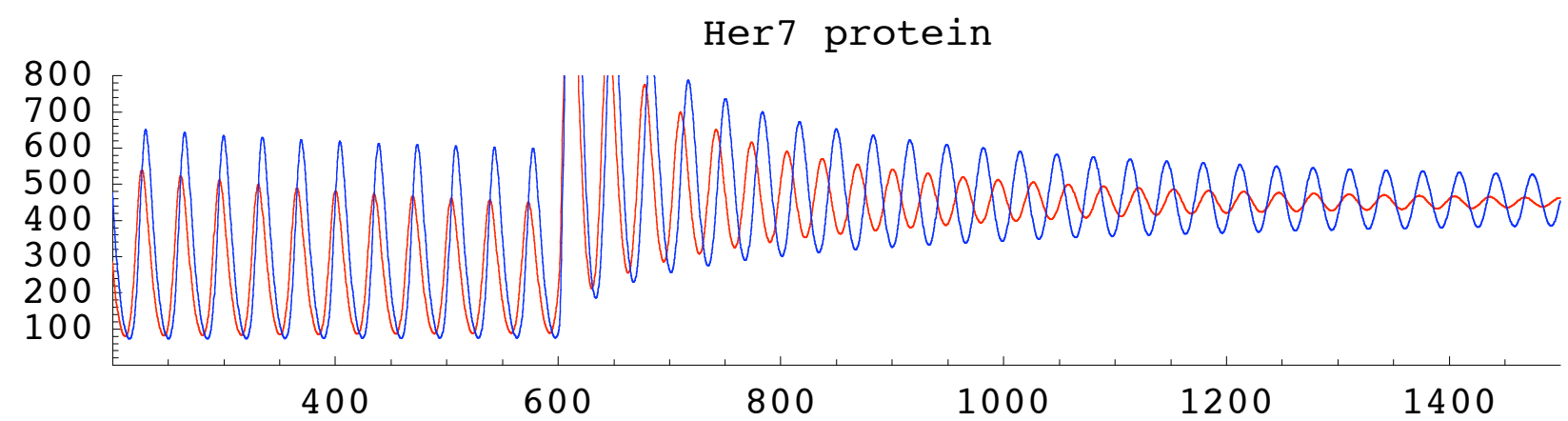
Her7 protein
800
700
600
500
400
300
200
100
400
600
800
1000
1200
1400
